# Identification of potential genetic risk factors for bipolar disorder by whole-exome sequencing

**DOI:** 10.1038/s41398-018-0291-7

**Published:** 2018-12-05

**Authors:** Thomas Husson, Jean-Baptiste Duboc, Olivier Quenez, Camille Charbonnier, Maud Rotharmel, Macarena Cuenca, Xavier Jegouzo, Anne-Claire Richard, Thierry Frebourg, Jean-François Deleuze, Anne Boland, Emmanuelle Genin, Stéphanie Debette, Christophe Tzourio, Dominique Campion, Gaël Nicolas, Olivier Guillin, The FREX Consortium

**Affiliations:** 1 Department of Research, Centre hospitalier du Rouvray, Sotteville-lès-Rouen, France; 2 Department of Genetics, Normandy Centre for Genomic and Personalized Medicine, Normandie Univ, UNIROUEN, Inserm U1245 and Rouen University Hospital, F 76000 Rouen, France; 3 Centre National de Recherche en Génomique Humaine, Institut de Génomique, CEA, Evry, France; 4 Inserm UMR-1078, CHRU Brest, Univ. Brest, Brest, France; 5 Univ. Bordeaux, Inserm, Bordeaux Population Health Research Center, UMR1219, F-33076 Bordeaux, France

## Abstract

This study aims at assessing the burden of rare (minor allele frequency < 1%) predicted damaging variants in the whole exome of 92 bipolar I disorder (BD) patients and 1051 controls of French ancestry. Patients exhibiting an extreme phenotype (earlier onset and family history of mood disorder) were preferentially included to increase the power to detect an association. A collapsing strategy was used to test the overall burden of rare variants in cases versus controls at the gene level. Only protein-truncating and predicted damaging missense variants were included in the analysis. Thirteen genes exhibited *p* values exceeding 10^−3^ and could be considered as potential risk factors for BD. Furthermore, the validity of the association was supported when the Exome Aggregation Consortium database non-Finnish European population was used as controls for eight of them. Their gene products are involved in various cerebral processes, some of which were previously implicated in BD and belong to pathways implicated in the therapeutic effect of lithium, the main mood stabilizer. However, exome-wide threshold for association study was not reached, emphasizing that larger samples are needed.

## Introduction

Bipolar I disorder (BD) is a chronic psychiatric illness characterized by mood oscillations, with episodes of mania and depression. The impact of BD on patients can be devastating, with up to 15% of patients committing suicide, enduring serious medical comorbidities such as endocrine disorders, cardiovascular disease, and drug abuse. The onset is usually during early adulthood. BD is known to be one of the leading cause of morbidity worldwide^1^. Family, twin, and adoption studies have provided strong evidence for the importance of genetic factors in the etiology of BD^2^. However, linkage studies in multiplex families identified mostly non-replicated findings^3^. Hence, Mendelian genes are unlikely to be involved in BD. Beside, in contrast with schizophrenia and autism, the burden of copy number variants seem not to be increased in BD^4^. More recently, genome-wide association studies (GWAS) identified several significantly associated loci carrying common variants that explain altogether only a small fraction of the genetic component of BD^5^. It has been hypothesized that for complex diseases such as BD, rare non-synonymous coding variants of moderate-to-large effect might explain a substantial part of the so-called missing heritability^6^. With the development and generalization of massive parallel sequencing, it is now possible to assess this hypothesis at the scale of all 20,000 human genes by whole-exome sequencing (WES). Concerning BD, this technology has already earn significant results in an intra-familial design^7^.

The power to detect associations of rare variants with a disease in a case–control setting is limited by the extreme rarity of most variants. To tackle this issue, variants can be collapsed at the gene-level or even gene networks. These strategies already provided significant results for several neuropsychiatric diseases such as Alzheimer disease at the gene level^8^, and schizophrenia at the gene-network level^9,10^.

This study aims at comparing the burden of rare predicted damaging variants in the whole exome between BD and controls at the gene level.

## Materials and methods

### Patients

Unrelated patients of French ancestry (*n* = 94) fulfilling the diagnosis of BD were recruited from Centre Hospitalier du Rouvray (*n* = 90) and from Centre Hospitalier Saint-Anne (*n* = 4). Patients were prescreened according to the MINI scale (Mini International Neuropsychiatric Interview) according to DSM-IV-TR criteria and underwent a comprehensive clinical examination including the assessment of the Diagnostic Interview for Genetic Studies scale^11^ and psychiatric family history through Affective Disorder Evaluation^12^. Patients were considered as lithium responders if the treatment had been administered for >2 years^13^.

We selected patients with a positive family history of affective disorder (bipolar disorder in one first degree relative and one in first to third-degree relative) and 63% of them had an early onset (before 22 years^14^). Under the assumption of stronger genetics factors involved in these subjects, this extreme phenotype sampling strategy aimed at increasing the statistical power^15^.

### Controls

A total of 1084 controls of French ancestry were recruited from two studies. A first series of 585 controls belongs to the FREX (French Exome) project, which aims at studying the stratification of rare variants among the French population and consists in healthy subjects recruited from six different cities. The remaining 499 belong from the three city cohort^16^, which includes elderly subjects not diagnosed with dementia.

All patients and controls gave informed, written consent for genetic analyses. This study was approved by the ethics committee of our institution.

### Whole-exome sequencing

Exomes were captured using the Agilent SureSelect Human All Exon Kit (Santa Clara, United-States) V5 or V5-UTR. Library preparation failed for two cases. Sequencing was performed in the remaining 92 cases on an Illumina HiSeq2500 (Illumina, San Diego, CA, USA) at the CNRGH (Centre National de Recherche en Génomique Humaine, Evry, France) with paired end mode, for 100 or 150 base pairs (bp) reads.

### Bioinformatics pipeline

Exome samples were all processed through the same bioinformatics pipeline following GATK 3.3-0 Best Practices recommendations and as previously described^8^. Reads were mapped to the GRCh37 1000Genomes build using BWA 0.7.5a^17^. Picard Tools 1.101 (http://picard.sourceforge.net) was used to flag duplicate reads. GATK^18^ was applied for short insertion and deletion (InDels) realignment, base quality score recalibration (BQSR) and finally single-nucleotide variants and InDel discovery using Haplotype Caller across all samples simultaneously. Variants were annotated with SnpEff 4.2^19^ and SnpSift 4.2^20^ software using dbNSFP 2.9.1 and Ensembl GRCh37.75. Exonic and splice site variants (located ± 2 bp around each coding exon) with a minor allele frequency (MAF) of  <1% in our whole data set were then extracted within each gene region. A quality score (VQSLOD) was estimated for each variant with the VQSR function from GATK 3.4. Only genotypes satisfying the following quality filters were retained for analysis: genotype read depth >6 and genotype quality  >20. Genotypes failing these two criteria were set as missing.

### Quality check

The sample QC was performed on the bi-allelic sites of the data set fulfilling the VQSLOD sensitivity threshold of 99.5% for single-nucleotide variants and 99% for InDels, as recommended in GATK best practices. To include multi-allelic sites in the analysis, they were converted into multiple bi-allelic variants that were then left-aligned using bcftools 1.3. Most checks were carried out with PLINK 1.9 (https://www.cog-genomics.org/plink2). All individuals in the sample were processed through the following steps: (i) verifying concordant sex information using Plink sex check, (ii) discarding contaminated samples identified as such by significantly high heterozygosity rates and freemix contamination scores provided by the VerifyBamID software, and (iii) discarding the sample of worst overall quality among each pair of samples with Plink pi_hat relatedness estimation exceeding 0.15 and (iv) discarding samples with overall missingness above 15%.

All cases and 1051 out of 1084 controls passed these quality checks.

Besides, no individual of divergent ancestry could be detected by either of the three following analyses: (i) extreme Pling neighbor statistics, (ii) principal component analyses on common (MAF > 5%) and rare (MAF < 5%) variants after exclusion of long-range linkage disequilibrium regions^21^ and variant pruning on linkage disequilibrium (*r*^2^ > 0.2), and (iii) outlying number of private mutations. Variants were excluded from statistical analysis if they (i) were missing in more than 5% of individuals, (ii) showed a significant deviation from Hardy–Weinberg equilibrium, (iii) presented significantly different missing call rates between cases and controls, as confirmed by Plink test missing at a threshold of 10^−6^, or (iv) showed an average allele balance below 25% or above 75% for heterozygous calls or below 90% for homozygous calls. We also excluded variants in low-complexity regions as identified by the mdust program^22^, and variants in simple tandem repeat regions located by Tandem Repeats Finder^23^ and retrieved using the UCSC Table browser^24^.

### Statistics

A standard collapsing approach was used to test the overall burden of rare variants in cases vs controls at the gene level. Protein-truncating variants (PTV) were defined following the annotation as “LOF” by SnpEff based on the conservative definition provided by MacArthur et al.^25^. In brief, they included the nonsense, frameshift InDels, and canonical splice site variants that are predicted to result in a loss of function, taking into account their position in the gene sequence. Missense variants were classified as Mis3, Mis2, and Mis1 or benign if they were predicted damaging by respectively 3, 2, 1, or 0 of the following bioinformatics predictions tools: polyphen2 (HumDiv)^26^, Mutation Taster2^27^, and SIFT^28^. After exclusion of benign variants, statistical analyses were based on four embedded classes of variants: PTV, PTV+Mis3, PTV+Mis3+Mis2, and PTV+Mis3+Mis2+Mis1.

For every gene, the proportions of variant carriers were compared between cases and controls using a Fisher exact test with the R statistical software (http://www.R-project.org/). The same gene-level association tests were applied to every coding region to extract all possible gene-level *p* values. A Bonferroni adjusted *p* < 2.5 × 10^−6^ was considered to be statistically significant on an exome-wide level given the theoretical number of genes tested. In addition, we computed a false discovery rate (FDR).

### Consistency with ExAC

All variants detected in non-Finnish European (NFE) individuals from the Exome Aggregation Consortium database (ExAC) were downloaded from the open access web resource http://exac.broad institute.org/. Variants with MAF below 1% and missingness below 20% within this population were retained for analysis. They were annotated for functional consequences with SnpEff 4.2 and SnpSift 4.2 and classified into PTV, Mis3, Mis2, Mis1, and benign variants exactly like all variants from our own data set. For each variant class of interest (PTV, PTV+Mis3, PTV+Mis3+Mis2, PTV+Mis3+Mis2+Mis1), allele counts were aggregated by gene (total allele count, TAC) and divided by the maximum number of individuals with allele information (ANmax) on this gene to obtain an approximation of the proportion of variant carriers in the NFE population. The validity of this approximation relies on the rarity of double carriers, which is a sound assumption considering the extreme rarity of most variants, but also presumes that coverage is close to uniform within a gene and that most samples with missing information will not carry any variant.

Two Fisher exact tests were then computed. For each gene and variant class, the proportion of case variant carriers in our data set was compared with the ratio TAC/ANmax. Interpretation of this test was subject to the comparison of the proportion of control variant carriers in our data set to the ratio TAC/ANmax. This second test in particular should help highlight potential false positive results stemming from putatively abnormally low variant detection within our controls.

## Results

A total of 92 WES of unrelated BD patients and 1051 WES of controls passed the quality criteria for subsequent association analysis. The demographic and clinical characteristics of the sample are summarized in Table 1a, b. Except for rapid cycling, which is at the lower-end (11%) of what is usually observed in epidemiologic studies, all clinical features affected a proportion of bipolar patients consistent with the literature. On average per subject, 22,425 variants mapping to the exons or the canonical splice sites (−2; +2) were called 749 were rare (MAF < 1%), from which 330 were classified as PTV, Mis1, Mis2, or Mis3 and included in the association test.

**Table 1a:** Demographic characteristics of the sample

	Cases	Controls
*N*	92	1051
% females	58.70%	57.60%
Mean age	48	74
(sd. range)	(15.5, 18–84)	(15,19–103)
Mean age of onset	23.9	/
(sd. range)	(11.7, 8–45)

**Table 1b:** Clinical characteristics of the cases

Clinical feature	*N* (%)
Lithium response	59/92 (64%)
Suicidal attempt	42/92 (45%)
Rapid cycling	10/92 (11%)
Psychotic symptoms	58/92 (63%)
Substance abuse	35/92 (38%)
Depressive polarity	18/92 (20%)
Manic polarity	20/92 (22%)

When collapsing PTV with missense variants falling into the three categories of Mis3, Mis2, or Mis1, no gene reached the exome-wide *p* value threshold of 2.5 × 10^−6^ nor FDR threshold of 10%. However, 13 genes exceeded a *p* value of 10^−3^ with odds ratios (OR) ranging from 3 to 23.7 for 10 of them, whereas three showed no variants in controls, hence having infinite ORs (Table 2). The association was essentially driven by missense variants. For each gene, the number of PTV was extremely low (Supplementary table 1) and their inclusion only marginally affected the *p* value (Supplementary table 2).

**Table 2:** Top-hits for rare variants (MAF ≤ 1%) burden tests

*N* variant carriers					
Gene	Category	Cases	Controls	OR (CI 95%)	*p* value
*CCDC171*	PTV+Mis3+Mis2+Mis1	11 (11.9%)	31 (2.9%)	4.4 (1.9–9.5)	2.7.10^−4^
*FAM19A3* ^*5*^	PTV+Mis3+Mis2+Mis1*	5 (5.4%)	4 (0.4%)	15 (3.1–76.8)	3.0.10^−4^
*TCF7L1* ^*3*^	PTV+Mis3+Mis2+Mis1	5 (5.4%)	4 (0.4%)	15 (3.1–76.8)	3.0.10^−4^
*BOC* ^*2*^	PTV+Mis3+Mis2+Mis1*	10 (10.8%)	26 (2.5%)	4.8 (2–10.7)	3.1.10^−4^
*MYO1E*	PTV+Mis3+Mis2*	10 (10.8%)	27 (2.6%)	4.6 (1.9–10.2)	4.0.10^−4^
*ACPP*	PTV+Mis3	3 (3.2%)	0	∞ (4.8–∞)	5.1.10^−4^
*PLCXD3* ^*2*^	PTV+Mis3*	3 (3.2%)	0	∞ (4.8–∞)	5.1.10^−4^
*NDUFAF2* ^*2*^	PTV+Mis3*	4 (3.7%)	2 (0.2%)	23.7 (3.3–263.9)	5.2.10^−4^
*VPS52*	PTV+Mis3*	5 (5.4%)	5 (0.5%)	12 (2.7–53.1)	5.5.10^−4^
*ERI3*	PTV+Mis3+Mis2+Mis1*	3 (3.2%)	0	∞ (4.8–∞)	5.1.10^−4^
*ARHGAP9* ^*1.4*^	PTV+Mis3+Mis2+Mis1	11 (11.9%)	36 (3.4%)	3.8 (1.7–8)	7.7.10^−4^
*ABCC10*	PTV+Mis3+Mis2+Mis1	15 (13.8%)	64 (6.1%)	3 (1.5–5.6)	9.2.10^−4^
*LGR5* ^*2*^	PTV+Mis3+Mis2	10 (10.8%)	31 (2.9%)	4 (1.7–8.7)	9.6.10^−4^

*OR (CI 95%)* odds ratio with 95% confidence interval, *PTV* protein-truncating variant, *Mis3* missense variants predicted damaging by 3 software out of 3, *Mis2* missense variants predicted damaging by 2 software out of 3, *Mis1* missense variants predicted damaging by 1 software out of 3. Genes previously implicated in (1) myelination, (2) neurodevelopment, (3) corticotropic axis, (4) microglia, (5) oxidative stress. * No PTV observed

Except for *ACPP*, all top-hits genes are expressed in the brain^29^. Nevertheless, an isoform of *ACPP* is known to be expressed in brain^30^.

To assess the plausibility of these results, we compared both the proportions of variant carriers among cases and controls with an approximation of the proportion of variant carriers observed in the ExAC NFE population through the TAC/ANmax ratio (Table 3). *NDUFAF2* gene showed a large depletion of PTV, Mis3, Mis2, and Mis1 missense variant carriers among our controls compared with what was observed in the ExAC NFE population, which suggests that the strength of the association might result from chance alone for this gene. To a lesser extent, two genes showed a relatively slight depletion in the same categories of variants in our controls compared with ExAC NFE data: *CCDC171* and *FAM19A3*. Of note, *LGR5* gene did not exhibit a clear depletion in PTV, Mis3, and Mis2 variants but the inclusion of Mis1 variants disclosed a significant depletion of variants in our controls. Hence, comparison with ExAC data also casted doubts about the reality or the strength of the association of *CCDC171*, *FAM19A3*, and *LGR5* variants with BD. On the contrary, *MYOE1* gene suffered from a lack of variants within the ExAC NFE population, not allowing us to compare cases to the ExAC NFE population.

**Table 3:** Comparison with ExAC data

		*N* variant carriers			Cases vs ExAC NFE		Controls vs ExAC NFE	
Gene	Category	Cases	Controls	ExAC NFE	OR ExAC NFE	*p* value	OR ExAC NFE	*p* value
*CCDC171* ^*2*^	PTV+Mis3+Mis2+Mis1	11 (11.9%)	31 (2.9%)	1347 (4%)	3.2 (1.5–6.1)	1.25×10^−3^	0.72 (0.49–1.04)	7.86×10^−2^
*FAM19A3* ^*2*^	PTV+Mis3+Mis2+Mis1*	5 (5.4%)	4 (0.4%)	362 (1.1%)	5.2 (1.6–12.8)	3.49×10^−3^	0.35 (0.09–0.90)	2.15×10^−2^
*TCF7L1* ^*1*^	PTV+Mis3+Mis2+Mis1	5 (5.4%)	4 (0.4%)	145 (0.4%)	13.2 (4.1–32.6)	6.08×10^−5^	0.88 (0.23–2.30)	1
*BOC* ^*1*^	PTV+Mis3+Mis2+Mis1*	10 (10.8%)	26 (2.5%)	651 (1.9%)	6.1 (2.8–11.9)	1.42×10^−5^	1.27 (0.82–1.90)	2.15×10−^1^
*MYO1* ^*4*^	PTV+Mis3+Mis2*	10 (10.8%)	27 (2.6%)	272 (0.8%)	14.8 (6.8–29.1)	6.05×10^−9^	3.21 (2.07–4.8)	6.24×10^−7^
*ACPP* ^*1*^	PTV+Mis3	3 (3.2%)	0	88 (0.3%)	12.7 (2.5–39.7)	2.05×10^−3^	0 (0–1.36)	1.18×10^−1^
*PLCXD3* ^*1*^	PTV+Mis3*	3 (3.2%)	0	39 (0.1%)	28.8 (5.6–93.3)	2.14×10^−4^	0 (0–3.15)	6.33×10^−1^
*NDUFAF2* ^*3*^	PTV+Mis3+Mis2+Mis1	4 (3.7%)	2 (0.2%)	434 (1.3%)	3.5 (0.9–9.2)	3.30×10^−2^	0.4 (0.02–0.53)	2.25×10^−4^
*VPS52* ^*1*^	PTV+Mis3*	5 (5.4%)	5 (0.5%)	121 (0.3%)	15.8 (4.9–39.3)	2.64×10^−5^	1.31 (0.42–3.16)	4.40×10^−1^
*ERI3* ^*1*^	PTV+Mis3+Mis2+Mis1*	3 (3.2%)	0	84 (0.2%)	13.4 (2.7–41.7)	1.80×10^−3^	0 (0–1.42)	1.88×10^−1^
*ARHGAP9* ^*1*^	PTV+Mis3+Mis2+Mis1	11 (11.9%)	36 (3.4%)	931 (2.7%)	4.7 (2.3–9)	5.59×10^−5^	1.24 (0.86–1.74)	2.17×10^−1^
*ABCC10* ^*1*^	PTV+Mis3+Mis2+Mis1	15 (13.8%)	64 (6.1%)	1604 (4.1%)	3.9 (2–6.8)	3.41×10^−5^	1.28 (0.98–1.66)	6.76×10^−2^
*LGR5* ^*2*^	PTV+Mis3+Mis2	10 (10.8%)	31 (2.9%)	1223 (3.6%)	3.2 (1.5–6.2)	2.08×10^−3^	0.80 (0.54–1.15)	2.42×10^−1^

*OR (CI 95%)* odds ratio with 95% confidence interval, *NFE* non-Finnish Europeans. Controls. Robustness of the association results (1) reinforced (2) questionable (3) potential false positive (4) not analyzable

In the light of ExAC, the remaining association results seem more robust. The absence of PTV, Mis3, Mis2, or Mis1 variants in our controls overestimated the strength of the association of *ACPP*, *ERI3*, and *PLCXD3* variants with BD in our data but appears to be consistent with the extremely low TAC/ANmax ratio in ExAC NFE data (Table 3). Regarding the other genes *TCF7L1*, *BOC*, *VPS52*, *ABCC10*, and *ARHGAP9*, comparison ExAC NFE controls displayed odd ratios of similar range, thus supporting our results.

## Discussion

We found 13 genes exhibiting a burden of rare truncating and missense predicted damaging variants with a level of association significance below 10^−3^ and ORs all above three in our case–control study of 92 BD patients and 1051 ethnically matched controls. However, none of the association signals reached the exome-wide *p* value threshold of 2.5 × 10^−6^ and a FDR threshold of 10%. Our series of patients was enriched in patients with an early onset (63% patients with AOO below 22) and all patients had a positive family history of mood disorder. Despite limited sample sizes, this extreme phenotype sampling strategy is likely to have enriched our sample in patients carrying rare variants with a moderate-to-high impact.

To further examine the top-hits, we performed the same burden tests using the NFE individuals of the ExAC sample as controls (around 33,300 subjects depending on the depth of coverage). Given potential population stratification or exome coverage biases, as well as the absence of individual-level allelic information allowing for exact carrier proportion computations, it is not possible to conduct meta- analyses or draw firm conclusions from these statistics based on ExAC. However, consistency with ExAC allele frequencies strengthens the validity of the association for eight different genes, namely *TCF7L1*, *BOC*, *VPS52*, *ABCC10, ARHGAP9, PLCXD3, ACPP*, and *ERI3*. This is, to our knowledge, the first case–control study using WES of patients and ethnically matched controls. These genes can be considered as potential risk factors for BD that warrants further consideration. Among these genes, four deserve a particular attention.

*PLCXD3* (Phosphatidylinositol Specific Phospholipase C X Domain Containing 3) C and ARHGAP9 (Rho GTPase Activating Protein 9) are involved in the phosphoinositide pathway, which is suspected to be implicated in the therapeutic effect of lithium, the main mood stabilizer^31^. *PLCXD3* encodes a phospholipase C and this locus was associated with early-onset BD though common variants in a GWAS study^32^.

*ARHGAP9* encodes a Rho GTPase with a binding site for various phosphoinositides^33^. Moreover, it seems to be involved in a co-inhibition loop with *GLI1* (GLI Family Zinc Finger 1)^34^ whose expression is necessary for the correct repartition of dopaminergic neurons in the midbrain^35^ and for the remyelinisation process of the cerebral stem cells^36^, a process that is enhanced by lithium exposure^37^.

*TCF7L1* (Transcription Factor 7 like 1) encodes a transcription factor involved in the WNT pathway^38^, which is critical for the therapeutic effect of lithium^39^. *TCF7L1* has been implicated in the development of the corticotropic axis in mice^40^. Of note, two variants mapped to the binding site of Catenin Beta-1 (a central messenger in the WNT pathway) in two cases: c.112 C > A, p. Leu38Met (Mis1) and c.190 G > C, p(Glu64Gln) (Mis3). This active site is crucial in the mediation of the WNT pathway signaling.

Regarding the *BOC* (Brother Of CDON) gene, it encodes a protein involved in early and late neurodevelopmental processes such as axonal guidance and synaptogenesis, respectively^41,42^. Interestingly, three patients carried ac.1031 G > A, p.Cys344Tyr Mis3 variant (and 1/1051 control), which is predicted to remove a cysteine residue involved in a disulfide bond in the extracellular domain of this transmembrane protein, further increasing the probable deleteriousness of this variant to the protein function. This variant is rare in ExAC with a MAF < 0.001 among NFE individuals.

As show in Table 2, 7 out of 13 top-hits are known to be involved in various cerebral functions and pathways, which show defects or atypical functioning in BD.

A substantial part of these hits could be truly positive as the strength of the signal increased while using ExAC NFE as controls. However, exome-wide threshold for association study was not reached in this study, emphasizing that much larger samples are needed. Nevertheless, those results obtained on a small sample of extreme cases are encouraging and underline the importance of case selection in genetic association studies.

## Supplementary material

Supplementary table 1

Supplementary table 2

Supplementary table 1

Supplementary table 2
