# Supplementary material for: Identification of potential genetic risk factors for bipolar disorder by whole-exome sequencing
Source: Transl Psychiatry. 2018 Dec 5;8:268. doi: 10.1038/s41398-018-0291-7 (PMC6281607; doi:10.1038/s41398-018-0291-7)
Supplement: Supplementary file 1 — Supplementary table 1 [file 41398_2018_291_MOESM1_ESM.pdf]

Sample	Status	Chr	Position	ID	Ref	Alt	Type	Gene	BaseChang
24640	0	15	59430501	chr15_594	G	T	Mis2	MYO1E	c.3146C>A
PMD-BEL-(	1	15	59430501	chr15_594	G	T	Mis2	MYO1E	c.3146C>A
21005	0	15	59430501	chr15_594	G	T	Mis2	MYO1E	c.3146C>A
PMD-VIG-(	1	15	59430501	chr15_594	G	T	Mis2	MYO1E	c.3146C>A
21545	0	15	59453379	chr15_594	C	T	Mis3	MYO1E	c.2678G>A
21213	0	15	59455458	chr15_594	T	C	Mis3	MYO1E	c.2525A>G
B00FWWL	0	15	59466086	chr15_594	C	A	Mis3	MYO1E	c.2193G>T
ROU-TRA-4	0	15	59480359	chr15_594	G	C	Mis3	MYO1E	c.1862C>G
ROU-VER-6	0	15	59480399	chr15_594	C	T	Mis2	MYO1E	c.1822G>A
20084	0	15	59497600	chr15_594	G	T	Mis2	MYO1E	c.1615C>A
PMD-LEG-(	1	15	59497600	chr15_594	G	T	Mis2	MYO1E	c.1615C>A
20785	0	15	59497621	chr15_594	C	T	Mis3	MYO1E	c.1594G>A
21035	0	15	59497622	chr15_594	G	C	Mis3	MYO1E	c.1593C>G
ROU-BID-4	0	15	59497622	chr15_594	G	C	Mis3	MYO1E	c.1593C>G
B00GG7M	0	15	59497622	chr15_594	G	C	Mis3	MYO1E	c.1593C>G
21552	0	15	59497622	chr15_594	G	C	Mis3	MYO1E	c.1593C>G
PMD-LAI-0	1	15	59497622	chr15_594	G	C	Mis3	MYO1E	c.1593C>G
B00G74C	0	15	59497622	chr15_594	G	C	Mis3	MYO1E	c.1593C>G
21632	0	15	59497622	chr15_594	G	C	Mis3	MYO1E	c.1593C>G
B00GG8K	0	15	59497622	chr15_594	G	C	Mis3	MYO1E	c.1593C>G
B00GQF5	0	15	59497622	chr15_594	G	C	Mis3	MYO1E	c.1593C>G
PMD-HER-	1	15	59497622	chr15_594	G	C	Mis3	MYO1E	c.1593C>G
B00G74L	0	15	59497622	chr15_594	G	C	Mis3	MYO1E	c.1593C>G
21154	0	15	59497622	chr15_594	G	C	Mis3	MYO1E	c.1593C>G
B00G748	0	15	59497622	chr15_594	G	C	Mis3	MYO1E	c.1593C>G
PMD-LEF-(	1	15	59497622	chr15_594	G	C	Mis3	MYO1E	c.1593C>G
20261	0	15	59497622	chr15_594	G	C	Mis3	MYO1E	c.1593C>G
PMD-BAU-	1	15	59497622	chr15_594	G	C	Mis3	MYO1E	c.1593C>G
PMD-FRE-(	1	15	59497622	chr15_594	G	C	Mis3	MYO1E	c.1593C>G
ROU-TIE-1	1	15	59497622	chr15_594	G	C	Mis3	MYO1E	c.1593C>G
PMD-DUC-	1	15	59510221	chr15_595	G	A	Mis3	MYO1E	c.976C>T
24640	0	15	59519746	chr15_595	T	C	Mis2	MYO1E	c.554A>G
PMD-BEL-(	1	15	59519746	chr15_595	T	C	Mis2	MYO1E	c.554A>G
21005	0	15	59519746	chr15_595	T	C	Mis2	MYO1E	c.554A>G
B00G756	0	15	59519746	chr15_595	T	C	Mis2	MYO1E	c.554A>G
22712	0	15	59519746	chr15_595	T	C	Mis2	MYO1E	c.554A>G
PMD-VIG-(	1	15	59519746	chr15_595	T	C	Mis2	MYO1E	c.554A>G
20431	0	15	59523987	chr15_595	C	T	Mis3	MYO1E	c.424G>A
21086	0	15	59548493	chr15_595	C	T	Mis3	MYO1E	c.322G>A
B00FWXI	0	15	59553624	chr15_595	C	T	Mis3	MYO1E	c.232G>A
ROU-BOB-	0	15	59564546	chr15_595	C	T	Mis2	MYO1E	c.106G>A

AAChange	Transcript	OtherTran	MAF	MAFContr	MAFCases	ExAC_ALL_	ExAC_NFE_	ExAC_SAS_	ExAC_AMF
p.Pro1049	NM_004998.3		0.001748	0.0009515	0.01087	0.0024	0.0019	0.0080	0.0021
p.Pro1049	NM_004998.3		0.001748	0.0009515	0.01087	0.0024	0.0019	0.0080	0.0021
p.Pro1049	NM_004998.3		0.001748	0.0009515	0.01087	0.0024	0.0019	0.0080	0.0021
p.Pro1049	NM_004998.3		0.001748	0.0009515	0.01087	0.0024	0.0019	0.0080	0.0021
p.Gly893A	NM_004998.3		0.0004371	0.0004757	0	0.0004	0.0006	0.0003	0.0004
p.Asp842G	NM_004998.3		0.0004371	0.0004757	0	NA	NA	NA	NA
p.Glu731A	NM_004998.3		0.0004374	0.0004762	0	1.648e-05	2.997e-05	0	0
p.Ala621G	NM_004998.3		0.0004371	0.0004757	0	NA	NA	NA	NA
p.Glu608Ly	NM_004998.3		0.0004371	0.0004757	0	NA	NA	NA	NA
p.Leu539M	NM_004998.3		0.0008741	0.0004757	0.005435	0.0010	0.0016	0	0.0011
p.Leu539M	NM_004998.3		0.0008741	0.0004757	0.005435	0.0010	0.0016	0	0.0011
p.Glu532Ly	NM_004998.3		0.0004371	0.0004757	0	NA	NA	NA	NA
p.Ile531M	NM_004998.3		0.007867	0.005709	0.03261	0.0069	0.0092	0.0019	0.0013
p.Ile531M	NM_004998.3		0.007867	0.005709	0.03261	0.0069	0.0092	0.0019	0.0013
p.Ile531M	NM_004998.3		0.007867	0.005709	0.03261	0.0069	0.0092	0.0019	0.0013
p.Ile531M	NM_004998.3		0.007867	0.005709	0.03261	0.0069	0.0092	0.0019	0.0013
p.Ile531M	NM_004998.3		0.007867	0.005709	0.03261	0.0069	0.0092	0.0019	0.0013
p.Ile531M	NM_004998.3		0.007867	0.005709	0.03261	0.0069	0.0092	0.0019	0.0013
p.Ile531M	NM_004998.3		0.007867	0.005709	0.03261	0.0069	0.0092	0.0019	0.0013
p.Ile531M	NM_004998.3		0.007867	0.005709	0.03261	0.0069	0.0092	0.0019	0.0013
p.Ile531M	NM_004998.3		0.007867	0.005709	0.03261	0.0069	0.0092	0.0019	0.0013
p.Ile531M	NM_004998.3		0.007867	0.005709	0.03261	0.0069	0.0092	0.0019	0.0013
p.Ile531M	NM_004998.3		0.007867	0.005709	0.03261	0.0069	0.0092	0.0019	0.0013
p.Ile531M	NM_004998.3		0.007867	0.005709	0.03261	0.0069	0.0092	0.0019	0.0013
p.Ile531M	NM_004998.3		0.007867	0.005709	0.03261	0.0069	0.0092	0.0019	0.0013
p.Ile531M	NM_004998.3		0.007867	0.005709	0.03261	0.0069	0.0092	0.0019	0.0013
p.Ile531M	NM_004998.3		0.007867	0.005709	0.03261	0.0069	0.0092	0.0019	0.0013
p.Ile531M	NM_004998.3		0.007867	0.005709	0.03261	0.0069	0.0092	0.0019	0.0013
p.Ile531M	NM_004998.3		0.007867	0.005709	0.03261	0.0069	0.0092	0.0019	0.0013
p.Ile531M	NM_004998.3		0.007867	0.005709	0.03261	0.0069	0.0092	0.0019	0.0013
p.Arg326T	NM_004998.3		0.0008741	0	0.005435	0.0003	0.0003	0.0012	0
p.Asp185G	NM_004998.3		0.002622	0.001903	0.01087	0.0025	0.0021	0.0091	0.0004
p.Asp185G	NM_004998.3		0.002622	0.001903	0.01087	0.0025	0.0021	0.0091	0.0004
p.Asp185G	NM_004998.3		0.002622	0.001903	0.01087	0.0025	0.0021	0.0091	0.0004
p.Asp185G	NM_004998.3		0.002622	0.001903	0.01087	0.0025	0.0021	0.0091	0.0004
p.Asp185G	NM_004998.3		0.002622	0.001903	0.01087	0.0025	0.0021	0.0091	0.0004
p.Asp185G	NM_004998.3		0.002622	0.001903	0.01087	0.0025	0.0021	0.0091	0.0004
p.Val142M	NM_004998.3		0.0004371	0.0004757	0	8.239e-06	1.499e-05	0	0
p.Val108Ile	NM_004998.3		0.0004371	0.0004757	0	8.241e-06	0	6.058e-05	0
p.Gly78Arg	NM_004998.3		0.000439	0.000478	0	8.247e-06	1.5e-05	0	0
p.Val36Me	NM_004998.3		0.0004371	0.0004757	0	2.471e-05	4.495e-05	0	0

ExAC_AFR_	ExAC_EAS_	ExAC_OTH	ExAC_FIN_	ExAC_max	SIFT	Polyphen2	Mutation_	pLI score	gTEX_Mea
9.612e-05	0	0.0066	0	0.0080	D	B	D	0.047978	6.97
9.612e-05	0	0.0066	0	0.0080	D	B	D	0.047978	6.97
9.612e-05	0	0.0066	0	0.0080	D	B	D	0.047978	6.97
9.612e-05	0	0.0066	0	0.0080	D	B	D	0.047978	6.97
0.0002	0	0	0	0.0006	D	D	D	0.047978	6.97
NA	NA	NA	NA	NA	D	D	D	0.047978	6.97
0	0	0	0	2.997e-05	D	D	D	0.047978	6.97
NA	NA	NA	NA	NA	D	D	D	0.047978	6.97
NA	NA	NA	NA	NA	T	D	D	0.047978	6.97
9.625e-05	0	0	0.0003	0.0016	T	P	D	0.047978	6.97
9.625e-05	0	0	0.0003	0.0016	T	P	D	0.047978	6.97
NA	NA	NA	NA	NA	D	D	D	0.047978	6.97
0.0019	0	0.0044	0.0239	0.0239	D	D	D	0.047978	6.97
0.0019	0	0.0044	0.0239	0.0239	D	D	D	0.047978	6.97
0.0019	0	0.0044	0.0239	0.0239	D	D	D	0.047978	6.97
0.0019	0	0.0044	0.0239	0.0239	D	D	D	0.047978	6.97
0.0019	0	0.0044	0.0239	0.0239	D	D	D	0.047978	6.97
0.0019	0	0.0044	0.0239	0.0239	D	D	D	0.047978	6.97
0.0019	0	0.0044	0.0239	0.0239	D	D	D	0.047978	6.97
0.0019	0	0.0044	0.0239	0.0239	D	D	D	0.047978	6.97
0.0019	0	0.0044	0.0239	0.0239	D	D	D	0.047978	6.97
0.0019	0	0.0044	0.0239	0.0239	D	D	D	0.047978	6.97
0.0019	0	0.0044	0.0239	0.0239	D	D	D	0.047978	6.97
0.0019	0	0.0044	0.0239	0.0239	D	D	D	0.047978	6.97
0.0019	0	0.0044	0.0239	0.0239	D	D	D	0.047978	6.97
0.0019	0	0.0044	0.0239	0.0239	D	D	D	0.047978	6.97
0.0019	0	0.0044	0.0239	0.0239	D	D	D	0.047978	6.97
0.0019	0	0.0044	0.0239	0.0239	D	D	D	0.047978	6.97
0.0019	0	0.0044	0.0239	0.0239	D	D	D	0.047978	6.97
0.0019	0	0.0044	0.0239	0.0239	D	D	D	0.047978	6.97
0.0003	0.0001	0	0	0.0012	D	D	D	0.047978	6.97
9.61e-05	0	0.0055	0.0002	0.0091	T	P	D	0.047978	6.97
9.61e-05	0	0.0055	0.0002	0.0091	T	P	D	0.047978	6.97
9.61e-05	0	0.0055	0.0002	0.0091	T	P	D	0.047978	6.97
9.61e-05	0	0.0055	0.0002	0.0091	T	P	D	0.047978	6.97
9.61e-05	0	0.0055	0.0002	0.0091	T	P	D	0.047978	6.97
9.61e-05	0	0.0055	0.0002	0.0091	T	P	D	0.047978	6.97
0	0	0	0	1.499e-05	D	D	D	0.047978	6.97
0	0	0	0	6.058e-05	D	D	D	0.047978	6.97
0	0	0	0	1.5e-05	D	D	D	0.047978	6.97
0	0	0	0	4.495e-05	T	D	D	0.047978	6.97

gTEX_mea	gTEX_mea	gTEX_Diff_Exp_brain/other
2.41	8.45	0.285
2.41	8.45	0.285
2.41	8.45	0.285
2.41	8.45	0.285
2.41	8.45	0.285
2.41	8.45	0.285
2.41	8.45	0.285
2.41	8.45	0.285
2.41	8.45	0.285
2.41	8.45	0.285
2.41	8.45	0.285
2.41	8.45	0.285
2.41	8.45	0.285
2.41	8.45	0.285
2.41	8.45	0.285
2.41	8.45	0.285
2.41	8.45	0.285
2.41	8.45	0.285
2.41	8.45	0.285
2.41	8.45	0.285
2.41	8.45	0.285
2.41	8.45	0.285
2.41	8.45	0.285
2.41	8.45	0.285
2.41	8.45	0.285
2.41	8.45	0.285
2.41	8.45	0.285
2.41	8.45	0.285
2.41	8.45	0.285
2.41	8.45	0.285
2.41	8.45	0.285
2.41	8.45	0.285
2.41	8.45	0.285
2.41	8.45	0.285
2.41	8.45	0.285
2.41	8.45	0.285
2.41	8.45	0.285
2.41	8.45	0.285
2.41	8.45	0.285
2.41	8.45	0.285
2.41	8.45	0.285

Sample	Status	Chr	Position	ID	Ref	Alt	Type
B00G7K1	0	12	71898384	chr12_71898	T	TC	PTV
PMis2-DEV-0!	1	12	71898416	chr12_71898	A	G	Mis2
20938	0	12	71898446	chr12_71898	C	A	Mis3
B00FWWI	0	12	71898446	chr12_71898	C	A	Mis3
21770	0	12	71918244	chr12_71918	A	G	Mis2
20487	0	12	71950407	chr12_71950	A	G	Mis2
ROU-AVE-144	0	12	71950407	chr12_71950	A	G	Mis2
PMis2-DUC-0	1	12	71950407	chr12_71950	A	G	Mis2
PMis2-LET-07	1	12	71950407	chr12_71950	A	G	Mis2
B00GG7Q	0	12	71950407	chr12_71950	A	G	Mis2
B00GG65	0	12	71965299	chr12_71965	T	G	Mis3
21461	0	12	71971766	chr12_71971	C	G	Mis3
PMis2-DEL-08	1	12	71971766	chr12_71971	C	G	Mis3
PMis2-LOG-0	1	12	71971766	chr12_71971	C	G	Mis3
PMis2-PHO-0	1	12	71971766	chr12_71971	C	G	Mis3
PMis2-VIL-08	1	12	71971766	chr12_71971	C	G	Mis3
PMis2-LEN-0!	1	12	71971766	chr12_71971	C	G	Mis3
B00FWXL	0	12	71971766	chr12_71971	C	G	Mis3
21884	0	12	71971766	chr12_71971	C	G	Mis3
ROU-DEN-46!	0	12	71972585	chr12_71972	G	A	Mis2
B00GG80	0	12	71972585	chr12_71972	G	A	Mis2
20905	0	12	71972585	chr12_71972	G	A	Mis2
B00FWXB	0	12	71972585	chr12_71972	G	A	Mis2
21160	0	12	71972585	chr12_71972	G	A	Mis2
B00G74N	0	12	71972585	chr12_71972	G	A	Mis2
B00FWXE	0	12	71972585	chr12_71972	G	A	Mis2
20421	0	12	71972585	chr12_71972	G	A	Mis2
20392	0	12	71972585	chr12_71972	G	A	Mis2
20197	0	12	71972585	chr12_71972	G	A	Mis2
PMis2-LEG-07	1	12	71972585	chr12_71972	G	A	Mis2
ROU-PER-543	0	12	71972607	chr12_71972	C	T	Mis3
B00GWAQ	0	12	71972674	chr12_71972	G	C	Mis3
PMis2-DAN-0	1	12	71972674	chr12_71972	G	C	Mis3
23410	0	12	71978131	chr12_71978	C	G	Mis3
20804	0	12	71978131	chr12_71978	C	G	Mis3
22036	0	12	71978131	chr12_71978	C	G	Mis3
21406	0	12	71978131	chr12_71978	C	G	Mis3
24066	0	12	71978131	chr12_71978	C	G	Mis3
21701	0	12	71978131	chr12_71978	C	G	Mis3
ROU-CLA-516	0	12	71978131	chr12_71978	C	G	Mis3
20455	0	12	71978131	chr12_71978	C	G	Mis3

Gene	BaseChange	AAChange	Transcript	OtherTranscr	MAF	MAFControls	MAFCases
LGR5	c.213-3dupC		NM_003667.	PTV:NR_1105	0.0004371	0.0004757	0
LGR5	c.235A>G	p.Ser79Gly	NM_003667.	Mis2:NM_00	0.0004371	0	0.005435
LGR5	c.265C>A	p.Leu89Ile	NM_003667.	Mis3:NM_00	0.0008741	0.0009515	0
LGR5	c.265C>A	p.Leu89Ile	NM_003667.	Mis3:NM_00	0.0008741	0.0009515	0
LGR5	c.343A>G	p.Ser115Gly	NM_003667.	Mis2:NM_00	0.0004371	0.0004757	0
LGR5	c.653A>G	p.His218Arg	NM_003667.	Mis2:NM_00	0.002185	0.001427	0.01087
LGR5	c.653A>G	p.His218Arg	NM_003667.	Mis2:NM_00	0.002185	0.001427	0.01087
LGR5	c.653A>G	p.His218Arg	NM_003667.	Mis2:NM_00	0.002185	0.001427	0.01087
LGR5	c.653A>G	p.His218Arg	NM_003667.	Mis2:NM_00	0.002185	0.001427	0.01087
LGR5	c.653A>G	p.His218Arg	NM_003667.	Mis2:NM_00	0.002185	0.001427	0.01087
LGR5	c.1076T>G	p.Leu359Arg	NM_003667.	Mis3:NM_00	0.0004398	0.0004789	0
LGR5	c.1270C>G	p.Leu424Val	NM_003667.	Mis3:NM_00	0.003506	0.001431	0.02717
LGR5	c.1270C>G	p.Leu424Val	NM_003667.	Mis3:NM_00	0.003506	0.001431	0.02717
LGR5	c.1270C>G	p.Leu424Val	NM_003667.	Mis3:NM_00	0.003506	0.001431	0.02717
LGR5	c.1270C>G	p.Leu424Val	NM_003667.	Mis3:NM_00	0.003506	0.001431	0.02717
LGR5	c.1270C>G	p.Leu424Val	NM_003667.	Mis3:NM_00	0.003506	0.001431	0.02717
LGR5	c.1270C>G	p.Leu424Val	NM_003667.	Mis3:NM_00	0.003506	0.001431	0.02717
LGR5	c.1270C>G	p.Leu424Val	NM_003667.	Mis3:NM_00	0.003506	0.001431	0.02717
LGR5	c.1270C>G	p.Leu424Val	NM_003667.	Mis3:NM_00	0.003506	0.001431	0.02717
LGR5	c.1282G>A	p.Asp428Asn	NM_003667.	Mis2:NM_00	0.004808	0.004757	0.005435
LGR5	c.1282G>A	p.Asp428Asn	NM_003667.	Mis2:NM_00	0.004808	0.004757	0.005435
LGR5	c.1282G>A	p.Asp428Asn	NM_003667.	Mis2:NM_00	0.004808	0.004757	0.005435
LGR5	c.1282G>A	p.Asp428Asn	NM_003667.	Mis2:NM_00	0.004808	0.004757	0.005435
LGR5	c.1282G>A	p.Asp428Asn	NM_003667.	Mis2:NM_00	0.004808	0.004757	0.005435
LGR5	c.1282G>A	p.Asp428Asn	NM_003667.	Mis2:NM_00	0.004808	0.004757	0.005435
LGR5	c.1282G>A	p.Asp428Asn	NM_003667.	Mis2:NM_00	0.004808	0.004757	0.005435
LGR5	c.1282G>A	p.Asp428Asn	NM_003667.	Mis2:NM_00	0.004808	0.004757	0.005435
LGR5	c.1282G>A	p.Asp428Asn	NM_003667.	Mis2:NM_00	0.004808	0.004757	0.005435
LGR5	c.1282G>A	p.Asp428Asn	NM_003667.	Mis2:NM_00	0.004808	0.004757	0.005435
LGR5	c.1282G>A	p.Asp428Asn	NM_003667.	Mis2:NM_00	0.004808	0.004757	0.005435
LGR5	c.1304C>T	p.Ser435Leu	NM_003667.	Mis3:NM_00	0.0004371	0.0004757	0
LGR5	c.1371G>C	p.Gln457His	NM_003667.	Mis3:NM_00	0.0008749	0.0004762	0.005435
LGR5	c.1371G>C	p.Gln457His	NM_003667.	Mis3:NM_00	0.0008749	0.0004762	0.005435
LGR5	c.2341C>G	p.Pro781Ala	NM_003667.	Mis3:NM_00	0.003497	0.003806	0
LGR5	c.2341C>G	p.Pro781Ala	NM_003667.	Mis3:NM_00	0.003497	0.003806	0
LGR5	c.2341C>G	p.Pro781Ala	NM_003667.	Mis3:NM_00	0.003497	0.003806	0
LGR5	c.2341C>G	p.Pro781Ala	NM_003667.	Mis3:NM_00	0.003497	0.003806	0
LGR5	c.2341C>G	p.Pro781Ala	NM_003667.	Mis3:NM_00	0.003497	0.003806	0
LGR5	c.2341C>G	p.Pro781Ala	NM_003667.	Mis3:NM_00	0.003497	0.003806	0
LGR5	c.2341C>G	p.Pro781Ala	NM_003667.	Mis3:NM_00	0.003497	0.003806	0
LGR5	c.2341C>G	p.Pro781Ala	NM_003667.	Mis3:NM_00	0.003497	0.003806	0

ExAC_ALL_AF	ExAC_NFE_AI	ExAC_SAS_AF	ExAC_AMR_A	ExAC_AFR_AI	ExAC_EAS_AF	ExAC_OTH_A	ExAC_FIN_AF
0.0004	0.0002	0.0020	8.64e-05	9.617e-05	0	0	0
8.236e-06	1.498e-05	0	0	0	0	0	0
6.59e-05	8.99e-05	0	8.651e-05	9.61e-05	0	0	0
6.59e-05	8.99e-05	0	8.651e-05	9.61e-05	0	0	0
NA	NA	NA	NA	NA	NA	NA	NA
0.0027	0.0041	0.0024	0.0003	0.0004	0	0.0011	0.0015
0.0027	0.0041	0.0024	0.0003	0.0004	0	0.0011	0.0015
0.0027	0.0041	0.0024	0.0003	0.0004	0	0.0011	0.0015
0.0027	0.0041	0.0024	0.0003	0.0004	0	0.0011	0.0015
0.0027	0.0041	0.0024	0.0003	0.0004	0	0.0011	0.0015
NA	NA	NA	NA	NA	NA	NA	NA
0.0074	0.0071	0.0202	0.0016	0.0011	0.0005	0.0143	0.0065
0.0074	0.0071	0.0202	0.0016	0.0011	0.0005	0.0143	0.0065
0.0074	0.0071	0.0202	0.0016	0.0011	0.0005	0.0143	0.0065
0.0074	0.0071	0.0202	0.0016	0.0011	0.0005	0.0143	0.0065
0.0074	0.0071	0.0202	0.0016	0.0011	0.0005	0.0143	0.0065
0.0074	0.0071	0.0202	0.0016	0.0011	0.0005	0.0143	0.0065
0.0074	0.0071	0.0202	0.0016	0.0011	0.0005	0.0143	0.0065
0.0074	0.0071	0.0202	0.0016	0.0011	0.0005	0.0143	0.0065
0.0051	0.0059	0.0008	0.0008	0.0009	0	0.0056	0.0280
0.0051	0.0059	0.0008	0.0008	0.0009	0	0.0056	0.0280
0.0051	0.0059	0.0008	0.0008	0.0009	0	0.0056	0.0280
0.0051	0.0059	0.0008	0.0008	0.0009	0	0.0056	0.0280
0.0051	0.0059	0.0008	0.0008	0.0009	0	0.0056	0.0280
0.0051	0.0059	0.0008	0.0008	0.0009	0	0.0056	0.0280
0.0051	0.0059	0.0008	0.0008	0.0009	0	0.0056	0.0280
0.0051	0.0059	0.0008	0.0008	0.0009	0	0.0056	0.0280
0.0051	0.0059	0.0008	0.0008	0.0009	0	0.0056	0.0280
0.0051	0.0059	0.0008	0.0008	0.0009	0	0.0056	0.0280
0.0051	0.0059	0.0008	0.0008	0.0009	0	0.0056	0.0280
0.0007	1.501e-05	0	0.0003	0.0070	0	0.0011	0
0.0007	0.0010	0.0002	0.0007	9.623e-05	0	0.0011	0
0.0007	0.0010	0.0002	0.0007	9.623e-05	0	0.0011	0
0.0065	0.0074	6.056e-05	0.0004	0.0002	0	0.0099	0.0417
0.0065	0.0074	6.056e-05	0.0004	0.0002	0	0.0099	0.0417
0.0065	0.0074	6.056e-05	0.0004	0.0002	0	0.0099	0.0417
0.0065	0.0074	6.056e-05	0.0004	0.0002	0	0.0099	0.0417
0.0065	0.0074	6.056e-05	0.0004	0.0002	0	0.0099	0.0417
0.0065	0.0074	6.056e-05	0.0004	0.0002	0	0.0099	0.0417
0.0065	0.0074	6.056e-05	0.0004	0.0002	0	0.0099	0.0417
0.0065	0.0074	6.056e-05	0.0004	0.0002	0	0.0099	0.0417

ExAC_maxPO	SIFT	Polyphen2_H	Mutation_tas	pLI score	gTEX_Mean_	gTEX_mean_l	gTEX_mean_
0.0020				1.14E-05	0.79	1.18	0.66
1.498e-05	D,D,D,D	P,P	N	1.14E-05	0.79	1.18	0.66
9.61e-05	D,D,D,D	D,P	D	1.14E-05	0.79	1.18	0.66
9.61e-05	D,D,D,D	D,P	D	1.14E-05	0.79	1.18	0.66
NA	D,D,D,D	B,B	D	1.14E-05	0.79	1.18	0.66
0.0041	D,T,D,D	B,B	D	1.14E-05	0.79	1.18	0.66
0.0041	D,T,D,D	B,B	D	1.14E-05	0.79	1.18	0.66
0.0041	D,T,D,D	B,B	D	1.14E-05	0.79	1.18	0.66
0.0041	D,T,D,D	B,B	D	1.14E-05	0.79	1.18	0.66
0.0041	D,T,D,D	B,B	D	1.14E-05	0.79	1.18	0.66
NA	D,D,D,D	D,D	D	1.14E-05	0.79	1.18	0.66
0.0202	D,D,D,D	D,D	D	1.14E-05	0.79	1.18	0.66
0.0202	D,D,D,D	D,D	D	1.14E-05	0.79	1.18	0.66
0.0202	D,D,D,D	D,D	D	1.14E-05	0.79	1.18	0.66
0.0202	D,D,D,D	D,D	D	1.14E-05	0.79	1.18	0.66
0.0202	D,D,D,D	D,D	D	1.14E-05	0.79	1.18	0.66
0.0202	D,D,D,D	D,D	D	1.14E-05	0.79	1.18	0.66
0.0202	D,D,D,D	D,D	D	1.14E-05	0.79	1.18	0.66
0.0202	D,D,D,D	D,D	D	1.14E-05	0.79	1.18	0.66
0.0280	T,T,T,T	D,D	D	1.14E-05	0.79	1.18	0.66
0.0280	T,T,T,T	D,D	D	1.14E-05	0.79	1.18	0.66
0.0280	T,T,T,T	D,D	D	1.14E-05	0.79	1.18	0.66
0.0280	T,T,T,T	D,D	D	1.14E-05	0.79	1.18	0.66
0.0280	T,T,T,T	D,D	D	1.14E-05	0.79	1.18	0.66
0.0280	T,T,T,T	D,D	D	1.14E-05	0.79	1.18	0.66
0.0280	T,T,T,T	D,D	D	1.14E-05	0.79	1.18	0.66
0.0280	T,T,T,T	D,D	D	1.14E-05	0.79	1.18	0.66
0.0280	T,T,T,T	D,D	D	1.14E-05	0.79	1.18	0.66
0.0280	T,T,T,T	D,D	D	1.14E-05	0.79	1.18	0.66
0.0280	T,T,T,T	D,D	D	1.14E-05	0.79	1.18	0.66
0.0070	D,D,D,D	P,P	D	1.14E-05	0.79	1.18	0.66
0.0011	D,D,D,D	D,P	D	1.14E-05	0.79	1.18	0.66
0.0011	D,D,D,D	D,P	D	1.14E-05	0.79	1.18	0.66
0.0417	D,D,D,T	D,D,.	D,D	1.14E-05	0.79	1.18	0.66
0.0417	D,D,D,T	D,D,.	D,D	1.14E-05	0.79	1.18	0.66
0.0417	D,D,D,T	D,D,.	D,D	1.14E-05	0.79	1.18	0.66
0.0417	D,D,D,T	D,D,.	D,D	1.14E-05	0.79	1.18	0.66
0.0417	D,D,D,T	D,D,.	D,D	1.14E-05	0.79	1.18	0.66
0.0417	D,D,D,T	D,D,.	D,D	1.14E-05	0.79	1.18	0.66
0.0417	D,D,D,T	D,D,.	D,D	1.14E-05	0.79	1.18	0.66
0.0417	D,D,D,T	D,D,.	D,D	1.14E-05	0.79	1.18	0.66

gTEX_Diff_Exp_brain/other
1.787
1.787
1.787
1.787
1.787
1.787
1.787
1.787
1.787
1.787
1.787
1.787
1.787
1.787
1.787
1.787
1.787
1.787
1.787
1.787
1.787
1.787
1.787
1.787
1.787
1.787
1.787
1.787
1.787
1.787
1.787
1.787
1.787
1.787
1.787
1.787
1.787
1.787
1.787
1.787
1.787

Sample	Status	Chr	Position	ID	Ref	Alt	Type
B00GWAV	0	12	57866472	chr12_57866	C	T	PTV
B00FWY5	0	12	57867410	chr12_57867	C	A	Mis2
B00G74O	0	12	57867885	chr12_57867	G	A	Mis3
B00G74G	0	12	57867885	chr12_57867	G	A	Mis3
ROU-MAN-65	0	12	57867885	chr12_57867	G	A	Mis3
21300	0	12	57867885	chr12_57867	G	A	Mis3
PMis2-SUB-0	1	12	57867885	chr12_57867	G	A	Mis3
B00GQD6	0	12	57867885	chr12_57867	G	A	Mis3
PMis2-SAN-0	1	12	57867885	chr12_57867	G	A	Mis3
B00GG8F	0	12	57867885	chr12_57867	G	A	Mis3
PMis2-BAT-0	1	12	57867885	chr12_57867	G	A	Mis3
PMis2-REU-0	1	12	57867885	chr12_57867	G	A	Mis3
23054	0	12	57867885	chr12_57867	G	A	Mis3
20521	0	12	57867926	chr12_57867	TC	T	PTV
B00FWWD	0	12	57868454	chr12_57868	C	T	Mis3
ROU-LEG-513	0	12	57869934	chr12_57869	G	A	Mis3
21348	0	12	57870391	chr12_57870	T	C	Mis3
22004	0	12	57871015	chr12_57871	C	T	Mis2
PMis2-VOY-0	1	12	57871049	chr12_57871	G	A	Mis2
B00G73X	0	12	57871049	chr12_57871	G	A	Mis2
B00G73O	0	12	57871247	chr12_57871	C	T	Mis1
B00GWAK	0	12	57871247	chr12_57871	C	T	Mis1
PMis2-HER-0	1	12	57871258	chr12_57871	C	T	Mis1
B00GG64	0	12	57871262	chr12_57871	G	A	Mis3
B00G73Z	0	12	57871313	chr12_57871	G	A	Mis3
B00G72X	0	12	57871321	chr12_57871	T	C	Mis1
B00G749	0	12	57871321	chr12_57871	T	C	Mis1
20256	0	12	57871367	chr12_57871	G	A	Mis3
21640	0	12	57871388	chr12_57871	G	A	Mis3
PMis2-BOH-0	1	12	57871388	chr12_57871	G	A	Mis3
21280	0	12	57871388	chr12_57871	G	A	Mis3
B00G725	0	12	57871388	chr12_57871	G	A	Mis3
20386	0	12	57871388	chr12_57871	G	A	Mis3
B00GQCR	0	12	57871388	chr12_57871	G	A	Mis3
20759	0	12	57871388	chr12_57871	G	A	Mis3
21093	0	12	57871388	chr12_57871	G	A	Mis3
PMis2-LEB-0	1	12	57871388	chr12_57871	G	A	Mis3
ROU-LER-448	0	12	57871388	chr12_57871	G	A	Mis3
PMis2-GLA-0	1	12	57871388	chr12_57871	G	A	Mis3
PMis2-BOC-0	1	12	57871388	chr12_57871	G	A	Mis3
PMis2-FOL-0	1	12	57872372	chr12_57872	C	T	Mis1
21099	0	12	57872372	chr12_57872	C	T	Mis1
24652	0	12	57872372	chr12_57872	C	T	Mis1
21794	0	12	57872487	chr12_57872	T	C	Mis1
B00G7JH	0	12	57872537	chr12_57872	G	A	Mis2
20453	0	12	57872892	chr12_57872	G	A	PTV
22898	0	12	57872955	chr12_57872	G	A	Mis3

Gene	BaseChange	AAChange	Transcript	OtherTranscr	MAF	MAFControls	MAFCases
ARHGAP9	c.2025-1G>A		NM_032496.	PTV:NM_001	0.0004371	0.0004757	0
ARHGAP9	c.2000G>T	p.Arg667Leu	NM_032496.	Mis2:NM_00	0.0004371	0.0004757	0
ARHGAP9	c.1858C>T	p.Arg620Trp	NM_032496.	Mis3:NM_00	0.004808	0.00333	0.02174
ARHGAP9	c.1858C>T	p.Arg620Trp	NM_032496.	Mis3:NM_00	0.004808	0.00333	0.02174
ARHGAP9	c.1858C>T	p.Arg620Trp	NM_032496.	Mis3:NM_00	0.004808	0.00333	0.02174
ARHGAP9	c.1858C>T	p.Arg620Trp	NM_032496.	Mis3:NM_00	0.004808	0.00333	0.02174
ARHGAP9	c.1858C>T	p.Arg620Trp	NM_032496.	Mis3:NM_00	0.004808	0.00333	0.02174
ARHGAP9	c.1858C>T	p.Arg620Trp	NM_032496.	Mis3:NM_00	0.004808	0.00333	0.02174
ARHGAP9	c.1858C>T	p.Arg620Trp	NM_032496.	Mis3:NM_00	0.004808	0.00333	0.02174
ARHGAP9	c.1858C>T	p.Arg620Trp	NM_032496.	Mis3:NM_00	0.004808	0.00333	0.02174
ARHGAP9	c.1858C>T	p.Arg620Trp	NM_032496.	Mis3:NM_00	0.004808	0.00333	0.02174
ARHGAP9	c.1858C>T	p.Arg620Trp	NM_032496.	Mis3:NM_00	0.004808	0.00333	0.02174
ARHGAP9	c.1858C>T	p.Arg620Trp	NM_032496.	Mis3:NM_00	0.004808	0.00333	0.02174
ARHGAP9	c.1816delG	p.Asp606fs	NM_032496.	PTV:NM_001	0.0004371	0.0004757	0
ARHGAP9	c.1684G>A	p.Gly562Arg	NM_032496.	Mis3:NM_00	0.0004371	0.0004757	0
ARHGAP9	c.1132C>T	p.Arg378Trp	NM_032496.	Mis3:NM_00	0.0004371	0.0004757	0
ARHGAP9	c.1007A>G	p.Gln336Arg	NM_032496.	Mis3:NM_00	0.0004371	0.0004757	0
ARHGAP9	c.794G>A	p.Arg265Lys	NM_032496.	Mis2:NM_00	0.0004371	0.0004757	0
ARHGAP9	c.760C>T	p.Pro254Ser	NM_032496.	Mis2:NM_00	0.0008741	0.0004757	0.005435
ARHGAP9	c.760C>T	p.Pro254Ser	NM_032496.	Mis2:NM_00	0.0008741	0.0004757	0.005435
ARHGAP9	c.751G>A	p.Glu251Lys	NM_032496.	Mis1:NM_00	0.0008741	0.0009515	0
ARHGAP9	c.751G>A	p.Glu251Lys	NM_032496.	Mis1:NM_00	0.0008741	0.0009515	0
ARHGAP9	c.740G>A	p.Arg247His	NM_032496.	Mis1:NM_00	0.0004371	0	0.005435
ARHGAP9	c.736C>T	p.Arg246Cys	NM_032496.	Mis3:NM_00	0.0004371	0.0004757	0
ARHGAP9	c.685C>T	p.Arg229Cys	NM_032496.	Mis3:NM_00	0.0004371	0.0004757	0
ARHGAP9	c.677A>G	p.Asn226Ser	NM_032496.	Mis1:NM_00	0.0008741	0.0009515	0
ARHGAP9	c.677A>G	p.Asn226Ser	NM_032496.	Mis1:NM_00	0.0008741	0.0009515	0
ARHGAP9	c.631C>T	p.Pro211Ser	NM_032496.	Mis3:NM_00	0.0004371	0.0004757	0
ARHGAP9	c.610C>T	p.Pro204Ser	NM_032496.	Mis3:NM_00	0.005245	0.003806	0.02174
ARHGAP9	c.610C>T	p.Pro204Ser	NM_032496.	Mis3:NM_00	0.005245	0.003806	0.02174
ARHGAP9	c.610C>T	p.Pro204Ser	NM_032496.	Mis3:NM_00	0.005245	0.003806	0.02174
ARHGAP9	c.610C>T	p.Pro204Ser	NM_032496.	Mis3:NM_00	0.005245	0.003806	0.02174
ARHGAP9	c.610C>T	p.Pro204Ser	NM_032496.	Mis3:NM_00	0.005245	0.003806	0.02174
ARHGAP9	c.610C>T	p.Pro204Ser	NM_032496.	Mis3:NM_00	0.005245	0.003806	0.02174
ARHGAP9	c.610C>T	p.Pro204Ser	NM_032496.	Mis3:NM_00	0.005245	0.003806	0.02174
ARHGAP9	c.610C>T	p.Pro204Ser	NM_032496.	Mis3:NM_00	0.005245	0.003806	0.02174
ARHGAP9	c.610C>T	p.Pro204Ser	NM_032496.	Mis3:NM_00	0.005245	0.003806	0.02174
ARHGAP9	c.610C>T	p.Pro204Ser	NM_032496.	Mis3:NM_00	0.005245	0.003806	0.02174
ARHGAP9	c.610C>T	p.Pro204Ser	NM_032496.	Mis3:NM_00	0.005245	0.003806	0.02174
ARHGAP9	c.610C>T	p.Pro204Ser	NM_032496.	Mis3:NM_00	0.005245	0.003806	0.02174
ARHGAP9	c.485G>A	p.Gly162Glu	NM_032496.	Mis1:NM_00	0.001311	0.0009515	0.005435
ARHGAP9	c.485G>A	p.Gly162Glu	NM_032496.	Mis1:NM_00	0.001311	0.0009515	0.005435
ARHGAP9	c.485G>A	p.Gly162Glu	NM_032496.	Mis1:NM_00	0.001311	0.0009515	0.005435
ARHGAP9	c.370A>G	p.Arg124Gly	NM_032496.	Mis1:NM_00	0.0004371	0.0004757	0
ARHGAP9	c.320C>T	p.Pro107Leu	NM_032496.	Mis2:NM_00	0.0004371	0.0004757	0
ARHGAP9	c.298C>T	p.Gln100*	NM_032496.	PTV:NM_001	0.0004371	0.0004757	0
ARHGAP9	c.235C>T	p.Pro79Ser	NM_032496.	Mis3:NM_00	0.0004371	0.0004757	0

ExAC_ALL_AF	ExAC_NFE_AI	ExAC_SAS_AF	ExAC_AMR_A	ExAC_AFR_AI	ExAC_EAS_AF	ExAC_OTH_A	ExAC_FIN_AF
NA	NA	NA	NA	NA	NA	NA	NA
NA	NA	NA	NA	NA	NA	NA	NA
0.0034	0.0051	0.0002	0.0019	0.0008	0	0.0034	0.0051
0.0034	0.0051	0.0002	0.0019	0.0008	0	0.0034	0.0051
0.0034	0.0051	0.0002	0.0019	0.0008	0	0.0034	0.0051
0.0034	0.0051	0.0002	0.0019	0.0008	0	0.0034	0.0051
0.0034	0.0051	0.0002	0.0019	0.0008	0	0.0034	0.0051
0.0034	0.0051	0.0002	0.0019	0.0008	0	0.0034	0.0051
0.0034	0.0051	0.0002	0.0019	0.0008	0	0.0034	0.0051
0.0034	0.0051	0.0002	0.0019	0.0008	0	0.0034	0.0051
0.0034	0.0051	0.0002	0.0019	0.0008	0	0.0034	0.0051
0.0034	0.0051	0.0002	0.0019	0.0008	0	0.0034	0.0051
0.0034	0.0051	0.0002	0.0019	0.0008	0	0.0034	0.0051
NA	NA	NA	NA	NA	NA	NA	NA
3.295e-05	4.495e-05	0	0	0	0	0	0.0002
NA	NA	NA	NA	NA	NA	NA	NA
NA	NA	NA	NA	NA	NA	NA	NA
6.134e-05	0.0001	0	0	0	0	0	0
6.249e-05	9.332e-05	0	0	9.756e-05	0	0	0
6.249e-05	9.332e-05	0	0	9.756e-05	0	0	0
0.0003	0.0003	0	0.0004	0.0006	0	0.0011	0
0.0003	0.0003	0	0.0004	0.0006	0	0.0011	0
9.113e-05	0.0001	0.0001	8.639e-05	0	0	0	0
9.112e-05	0.0001	0	0.0002	0	0	0	0
3.318e-05	6.037e-05	0	0	0	0	0	0
8.302e-06	1.511e-05	0	0	0	0	0	0
8.302e-06	1.511e-05	0	0	0	0	0	0
NA	NA	NA	NA	NA	NA	NA	NA
0.0027	0.0040	0.0016	0.0010	0.0005	0	0.0023	0.0012
0.0027	0.0040	0.0016	0.0010	0.0005	0	0.0023	0.0012
0.0027	0.0040	0.0016	0.0010	0.0005	0	0.0023	0.0012
0.0027	0.0040	0.0016	0.0010	0.0005	0	0.0023	0.0012
0.0027	0.0040	0.0016	0.0010	0.0005	0	0.0023	0.0012
0.0027	0.0040	0.0016	0.0010	0.0005	0	0.0023	0.0012
0.0027	0.0040	0.0016	0.0010	0.0005	0	0.0023	0.0012
0.0027	0.0040	0.0016	0.0010	0.0005	0	0.0023	0.0012
0.0027	0.0040	0.0016	0.0010	0.0005	0	0.0023	0.0012
0.0027	0.0040	0.0016	0.0010	0.0005	0	0.0023	0.0012
0.0027	0.0040	0.0016	0.0010	0.0005	0	0.0023	0.0012
0.0027	0.0040	0.0016	0.0010	0.0005	0	0.0023	0.0012
0.0004	0.0006	0	0.0006	9.619e-05	0	0	0
0.0004	0.0006	0	0.0006	9.619e-05	0	0	0
0.0004	0.0006	0	0.0006	9.619e-05	0	0	0
2.475e-05	3.003e-05	0	0	9.643e-05	0	0	0
0.0002	0.0003	0.0002	0.0006	0	0	0	0.0002
4.944e-05	8.996e-05	0	0	0	0	0	0
NA	NA	NA	NA	NA	NA	NA	NA

ExAC_maxPO	SIFT	Polyphen2_H	Mutation_tas	pLI score	gTEX_Mean_	gTEX_mean_l	gTEX_mean_
NA			D	2.89E-09	6.51	1.44	8.16
NA	.,D,D,D,D,D,D	.,P,P,B	N,N	2.89E-09	6.51	1.44	8.16
0.0051	D,D,D,D,D,D,I	D,D,D,D,D	D	2.89E-09	6.51	1.44	8.16
0.0051	D,D,D,D,D,D,I	D,D,D,D,D	D	2.89E-09	6.51	1.44	8.16
0.0051	D,D,D,D,D,D,I	D,D,D,D,D	D	2.89E-09	6.51	1.44	8.16
0.0051	D,D,D,D,D,D,I	D,D,D,D,D	D	2.89E-09	6.51	1.44	8.16
0.0051	D,D,D,D,D,D,I	D,D,D,D,D	D	2.89E-09	6.51	1.44	8.16
0.0051	D,D,D,D,D,D,I	D,D,D,D,D	D	2.89E-09	6.51	1.44	8.16
0.0051	D,D,D,D,D,D,I	D,D,D,D,D	D	2.89E-09	6.51	1.44	8.16
0.0051	D,D,D,D,D,D,I	D,D,D,D,D	D	2.89E-09	6.51	1.44	8.16
0.0051	D,D,D,D,D,D,I	D,D,D,D,D	D	2.89E-09	6.51	1.44	8.16
0.0051	D,D,D,D,D,D,I	D,D,D,D,D	D	2.89E-09	6.51	1.44	8.16
0.0051	D,D,D,D,D,D,I	D,D,D,D,D	D	2.89E-09	6.51	1.44	8.16
NA				2.89E-09	6.51	1.44	8.16
0.0002	D,D,D,D,D,D,I	D,D,D,D,D	D	2.89E-09	6.51	1.44	8.16
NA	D,D,D,D,D,D,I	D,D,D,D,D	D	2.89E-09	6.51	1.44	8.16
NA	D,D,D,D,D,D,I	P,B,B,B,B	D	2.89E-09	6.51	1.44	8.16
0.0001	T,T,D,T,T,T,T,	D,D,D,D,P,B	N	2.89E-09	6.51	1.44	8.16
9.756e-05	T,T,T,T,T,T,T,T	D,B,P,P,P,B	D	2.89E-09	6.51	1.44	8.16
9.756e-05	T,T,T,T,T,T,T,T	D,B,P,P,P,B	D	2.89E-09	6.51	1.44	8.16
0.0011	T,T,T,T,T,T,T,T	P,B,B,B,B,B	N	2.89E-09	6.51	1.44	8.16
0.0011	T,T,T,T,T,T,T,T	P,B,B,B,B,B	N	2.89E-09	6.51	1.44	8.16
0.0001	T,T,T,T,T,T,T,T	B,B,B,B,B,B	D	2.89E-09	6.51	1.44	8.16
0.0002	D,D,D,D,D,D,I	B,B,D,B,D,D	D	2.89E-09	6.51	1.44	8.16
6.037e-05	D,D,D,D,D,D,I	D,D,D,D,D,D	D	2.89E-09	6.51	1.44	8.16
1.511e-05	T,T,T,T,T,T,T,T	B,B,B,B,B,B	D	2.89E-09	6.51	1.44	8.16
1.511e-05	T,T,T,T,T,T,T,T	B,B,B,B,B,B	D	2.89E-09	6.51	1.44	8.16
NA	T,T,T,T,T,T,T,I	P,B,P,B,B,B	D	2.89E-09	6.51	1.44	8.16
0.0040	T,T,T,T,T,D,D,	D,P,D,P,P,P	D	2.89E-09	6.51	1.44	8.16
0.0040	T,T,T,T,T,D,D,	D,P,D,P,P,P	D	2.89E-09	6.51	1.44	8.16
0.0040	T,T,T,T,T,D,D,	D,P,D,P,P,P	D	2.89E-09	6.51	1.44	8.16
0.0040	T,T,T,T,T,D,D,	D,P,D,P,P,P	D	2.89E-09	6.51	1.44	8.16
0.0040	T,T,T,T,T,D,D,	D,P,D,P,P,P	D	2.89E-09	6.51	1.44	8.16
0.0040	T,T,T,T,T,D,D,	D,P,D,P,P,P	D	2.89E-09	6.51	1.44	8.16
0.0040	T,T,T,T,T,D,D,	D,P,D,P,P,P	D	2.89E-09	6.51	1.44	8.16
0.0040	T,T,T,T,T,D,D,	D,P,D,P,P,P	D	2.89E-09	6.51	1.44	8.16
0.0040	T,T,T,T,T,D,D,	D,P,D,P,P,P	D	2.89E-09	6.51	1.44	8.16
0.0040	T,T,T,T,T,D,D,	D,P,D,P,P,P	D	2.89E-09	6.51	1.44	8.16
0.0040	T,T,T,T,T,D,D,	D,P,D,P,P,P	D	2.89E-09	6.51	1.44	8.16
0.0040	T,T,T,T,T,D,D,	D,P,D,P,P,P	D	2.89E-09	6.51	1.44	8.16
0.0006	T,T,T,T,T,T	B,B,P,B,B	N	2.89E-09	6.51	1.44	8.16
0.0006	T,T,T,T,T,T	B,B,P,B,B	N	2.89E-09	6.51	1.44	8.16
0.0006	T,T,T,T,T,T	B,B,P,B,B	N	2.89E-09	6.51	1.44	8.16
9.643e-05	T,T,T,T,T,D	B,B,B,B,B	N	2.89E-09	6.51	1.44	8.16
0.0006	D,D,D,D,D,D	P,P,P,P,P	N	2.89E-09	6.51	1.44	8.16
8.996e-05			A	2.89E-09	6.51	1.44	8.16
NA	D,D,D,D,D	D,D,D,D,D	D	2.89E-09	6.51	1.44	8.16

gTEX_Diff_Exp_brain/other
0.177
0.177
0.177
0.177
0.177
0.177
0.177
0.177
0.177
0.177
0.177
0.177
0.177
0.177
0.177
0.177
0.177
0.177
0.177
0.177
0.177
0.177
0.177
0.177
0.177
0.177
0.177
0.177
0.177
0.177
0.177
0.177
0.177
0.177
0.177
0.177
0.177
0.177
0.177
0.177
0.177
0.177
0.177
0.177
0.177
0.177
0.177

Sample	Status	Chr	Position	ID	Ref	Alt	Type
PMD-OUI-083	1	1	44804955	chr1_448049	T	C	MD
PMD-SOU-07	1	1	44820629	chr1_448206	G	A	SD
PMD-NOT-09	1	1	44820667	chr1_448206	C	G	SD

Gene	BaseChange	AAChange	Transcript	OtherTranscr	MAF	MAFControls	MAFCases
ERI3	c.251A>G	p.Gln84Arg	NM_024066.[illegible]	MD:NM_001[illegible]	0.0004417	0	0.005435
ERI3	c.70C>T	p.Pro24Ser	NM_024066.2		0.0004371	0	0.005435
ERI3	c.32G>C	p.Gly11Ala	NM_024066.2		0.0004371	0	0.005435

ExAC_ALL_AF	ExAC_NFE_AI	ExAC_SAS_AF	ExAC_AMR_A	ExAC_AFR_AI	ExAC_EAS_AF	ExAC_OTH_A	ExAC_FIN_AF
NA	NA	NA	NA	NA	NA	NA	NA
0.0006	0.0010	0	0	0.0003	0	0	0
NA	NA	NA	NA	NA	NA	NA	NA

ExAC_maxPO	SIFT	Polyphen2_H	Mutation_tas	pLI score	gTEX_Mean_	gTEX_mean_	gTEX_mean_
NA	D,D	B,B,B	D	0.12975609	17.74	25.28	15.29
0.0010	D,T,T,D	P,P	D	0.12975609	17.74	25.28	15.29
NA	D,D,T,D	D,D	D	0.12975609	17.74	25.28	15.29

gTEX_Diff_Exp_brain/other
1.653
1.653
1.653

Sample	Status	Chr	Position	ID	Ref	Alt	Type
PMis2-SOU-0	1	1	113264968	chr1_113264	C	T	Mis2
20469	0	1	113264968	chr1_113264	C	T	Mis2
PMis2-LEG-07	1	1	113264968	chr1_113264	C	T	Mis2
B00FWWI	0	1	113264968	chr1_113264	C	T	Mis2
21159	0	1	113266594	chr1_113266	G	A	Mis1
PMis2-LOG-0	1	1	113266594	chr1_113266	G	A	Mis1
PMis2-FLA-08	1	1	113266636	chr1_113266	C	T	Mis2
B00G72W	0	1	113266716	chr1_113266	C	T	Mis3
PMis2-BOR-0	1	1	113269294	chr1_113269	C	T	Mis1

Gene	BaseChange	AAChange	Transcript	OtherTranscr	MAF	MAFControls	MAFCases
FAM19A3	c.113C>T	p.Thr38Ile	NM_0010044	Mis2:NM_18	0.001748	0.0009515	0.01087
FAM19A3	c.113C>T	p.Thr38Ile	NM_0010044	Mis2:NM_18	0.001748	0.0009515	0.01087
FAM19A3	c.113C>T	p.Thr38Ile	NM_0010044	Mis2:NM_18	0.001748	0.0009515	0.01087
FAM19A3	c.113C>T	p.Thr38Ile	NM_0010044	Mis2:NM_18	0.001748	0.0009515	0.01087
FAM19A3	c.293G>A	p.Arg98His	NM_001004440.1		0.0008757	0.0004766	0.005435
FAM19A3	c.293G>A	p.Arg98His	NM_001004440.1		0.0008757	0.0004766	0.005435
FAM19A3	c.335C>T	p.Pro112Leu	NM_0010044	SYNONYMOU	0.0004371	0	0.005435
FAM19A3	c.415C>T	p.Arg139Trp	NM_0010044	Mis3:NM_18	0.0004425	0.0004822	0
FAM19A3	c.502C>T	p.Leu168Phe	NM_001004440.1		0.0004371	0	0.005435

ExAC_ALL_AF	ExAC_NFE_AI	ExAC_SAS_AF	ExAC_AMR_A	ExAC_AFR_AI	ExAC_EAS_AF	ExAC_OTH_A	ExAC_FIN_AF
0.0016	0.0004	0	0.0003	0.0165	0	0.0022	0
0.0016	0.0004	0	0.0003	0.0165	0	0.0022	0
0.0016	0.0004	0	0.0003	0.0165	0	0.0022	0
0.0016	0.0004	0	0.0003	0.0165	0	0.0022	0
0.0014	0.0023	0.0001	0.0005	0.0009	0	0	0.0002
0.0014	0.0023	0.0001	0.0005	0.0009	0	0	0.0002
NA	NA	NA	NA	NA	NA	NA	NA
0.0002	0.0002	0	9.24e-05	0.0004	0.0001	0	0
0.0014	0.0012	0	0	0	0	0.0022	0.0125

ExAC_maxPO	SIFT	Polyphen2_H	Mutation_tas	pLI score	gTEX_Mean_	gTEX_mean_	gTEX_mean_
0.0165	T,T	B,P	D	0.02714738	0.3	0.55	0.22
0.0165	T,T	B,P	D	0.02714738	0.3	0.55	0.22
0.0165	T,T	B,P	D	0.02714738	0.3	0.55	0.22
0.0165	T,T	B,P	D	0.02714738	0.3	0.55	0.22
0.0023	D	B	N	0.02714738	0.3	0.55	0.22
0.0023	D	B	N	0.02714738	0.3	0.55	0.22
NA	D	B	D	0.02714738	0.3	0.55	0.22
0.0004	D,D	D,D	D,D	0.02714738	0.3	0.55	0.22
0.0125	D	B	N	0.02714738	0.3	0.55	0.22

gTEX_Diff_Exp_brain/other
2.445
2.445
2.445
2.445
2.445
2.445
2.445
2.445
2.445

Sample	Status	Chr	Position	ID	Ref	Alt	Type
PMis2-LEN-09	1	2	85360919	chr2_853609	C	A	Mis1
PMis2-BON-0	1	2	85360997	chr2_853609	G	C	Mis3
PMis2-VIL-08	1	2	85361512	chr2_853615	TC	T	PTV
21930	0	2	85531044	chr2_855310	G	A	Mis3
21783	0	2	85531044	chr2_855310	G	A	Mis3
B00G74P	0	2	85532411	chr2_855324	A	G	Mis2
PMis2-DRO-0	1	2	85536202	chr2_855362	G	A	Mis3
PMis2-BOU-0	1	2	85536322	chr2_855363	C	T	Mis3
ROU-LEB-171	0	2	85536508	chr2_855365	G	A	Mis1

Gene	BaseChange	AAChange	Transcript	OtherTranscr	MAF	MAFControls	MAFCases
TCF7L1	c.112C>A	p.Leu38Met	NM_031283.2		0.0004371	0	0.005435
TCF7L1	c.190G>C	p.Glu64Gln	NM_031283.2		0.0004374	0	0.005435
TCF7L1	c.383delC	p.Pro128fs	NM_031283.2		0.0004371	0	0.005435
TCF7L1	c.685G>A	p.Glu229Lys	NM_031283.2		0.0008741	0.0009515	0
TCF7L1	c.685G>A	p.Glu229Lys	NM_031283.2		0.0008741	0.0009515	0
TCF7L1	c.874A>G	p.Met292Val	NM_031283.2		0.0004371	0.0004757	0
TCF7L1	c.1384G>A	p.Glu462Lys	NM_031283.2		0.0004371	0	0.005435
TCF7L1	c.1504C>T	p.Pro502Ser	NM_031283.2		0.0004371	0	0.005435
TCF7L1	c.1690G>A	p.Ala564Thr	NM_031283.2		0.0004371	0.0004757	0

ExAC_ALL_AF	ExAC_NFE_AI	ExAC_SAS_AF	ExAC_AMR_A	ExAC_AFR_AI	ExAC_EAS_AF	ExAC_OTH_A	ExAC_FIN_AF
0.0005	0.0011	0	0	0	0	0	0
NA	NA	NA	NA	NA	NA	NA	NA
NA	NA	NA	NA	NA	NA	NA	NA
NA	NA	NA	NA	NA	NA	NA	NA
NA	NA	NA	NA	NA	NA	NA	NA
6.601e-05	0.0001	0	8.642e-05	0	0	0	0
1.67e-05	0	0	0	0	0	0	0.0003
1.653e-05	1.505e-05	0	8.652e-05	0	0	0	0
0.0002	1.619e-05	8.256e-05	0.0003	0.0018	0	0.0013	0

ExAC_maxPO	SIFT	Polyphen2_H	Mutation_tas	pLI score	gTEX_Mean_	gTEX_mean_	gTEX_mean_
0.0011	T	B	D	0.75375201	8.14	2.08	10.11
NA	D	D	D	0.75375201	8.14	2.08	10.11
NA				0.75375201	8.14	2.08	10.11
NA	D	D	D	0.75375201	8.14	2.08	10.11
NA	D	D	D	0.75375201	8.14	2.08	10.11
0.0001	D	B	D	0.75375201	8.14	2.08	10.11
0.0003	D	P	D	0.75375201	8.14	2.08	10.11
8.652e-05	D	P	D	0.75375201	8.14	2.08	10.11
0.0018	T	B	D	0.75375201	8.14	2.08	10.11

gTEX_Diff_Exp_brain/other
0.206
0.206
0.206
0.206
0.206
0.206
0.206
0.206
0.206

Sample	Status	Chr	Position	ID	Ref	Alt	Type
21101	0	3	112969725	chr3_112969	C	A	Mis1
22172	0	3	112987262	chr3_112987	G	A	Mis3
B00GG64	0	3	112987271	chr3_112987	G	A	Mis2
B00FWWL	0	3	112991434	chr3_112991	G	A	Mis2
PMis2-LEG-0	1	3	112991493	chr3_112991	G	A	Mis1
22344	0	3	112991493	chr3_112991	G	A	Mis1
21024	0	3	112991493	chr3_112991	G	A	Mis1
PMis2-BAT-0	1	3	112991493	chr3_112991	G	A	Mis1
B00G73S	0	3	112991493	chr3_112991	G	A	Mis1
B00G7LM	0	3	112991493	chr3_112991	G	A	Mis1
B00G750	0	3	112991493	chr3_112991	G	A	Mis1
PMis2-DAN-0	1	3	112991514	chr3_112991	G	A	Mis2
B00GG8E	0	3	112991918	chr3_112991	C	T	Mis3
PMis2-SOU-0	1	3	112991985	chr3_112991	G	A	Mis3
PMis2-LEG-0	1	3	112991985	chr3_112991	G	A	Mis3
20214	0	3	112991985	chr3_112991	G	A	Mis3
PMis2-LAF-0	1	3	112991985	chr3_112991	G	A	Mis3
23064	0	3	112991994	chr3_112991	G	A	Mis1
22462	0	3	112992062	chr3_112992	C	T	Mis3
21490	0	3	112992072	chr3_112992	G	A	Mis1
23357	0	3	112992072	chr3_112992	G	A	Mis1
B00FWX9	0	3	112992140	chr3_112992	G	C	Mis1
B00GG6E	0	3	112992140	chr3_112992	G	C	Mis1
PMis2-DEV-0	1	3	112992140	chr3_112992	G	C	Mis1
22319	0	3	112993492	chr3_112993	G	A	Mis1
22357	0	3	112993501	chr3_112993	T	C	Mis3
B00FWYX	0	3	112997066	chr3_112997	C	T	Mis2
PMis2-DES-0	1	3	112998721	chr3_112998	G	A	Mis2
20625	0	3	112999432	chr3_112999	C	T	Mis3
B00G7KK	0	3	112999880	chr3_112999	G	T	Mis3
B00G7L6	0	3	112999967	chr3_112999	G	A	Mis3
22260	0	3	113002396	chr3_113002	T	C	Mis3
21101	0	3	113002475	chr3_113002	G	C	Mis2
PMis2-JOU-0	1	3	113003226	chr3_113003	T	C	Mis2
PMis2-COU-0	1	3	113003310	chr3_113003	C	T	Mis2
B00G7KT	0	3	113003311	chr3_113003	G	A	Mis1
22813	0	3	113003639	chr3_113003	C	T	Mis1

Gene	BaseChange	AAChange	Transcript	OtherTranscr	MAF	MAFControls	MAFCases
BOC	c.421C>A	p.His141Asn	NM_001301867.1		0.0004371	0.0004757	0
BOC	c.493G>A	p.Val165Ile	NM_0013018	Mis3:NM_03	0.0004371	0.0004757	0
BOC	c.502G>A	p.Glu168Lys	NM_0013018	Mis2:NM_03	0.0004371	0.0004757	0
BOC	c.845G>A	p.Ser282Asn	NM_0013018	Mis2:NM_03	0.0004371	0.0004757	0
BOC	c.904G>A	p.Asp302Asn	NM_0013018	Mis1:NM_03	0.003059	0.002379	0.01087
BOC	c.904G>A	p.Asp302Asn	NM_0013018	Mis1:NM_03	0.003059	0.002379	0.01087
BOC	c.904G>A	p.Asp302Asn	NM_0013018	Mis1:NM_03	0.003059	0.002379	0.01087
BOC	c.904G>A	p.Asp302Asn	NM_0013018	Mis1:NM_03	0.003059	0.002379	0.01087
BOC	c.904G>A	p.Asp302Asn	NM_0013018	Mis1:NM_03	0.003059	0.002379	0.01087
BOC	c.904G>A	p.Asp302Asn	NM_0013018	Mis1:NM_03	0.003059	0.002379	0.01087
BOC	c.904G>A	p.Asp302Asn	NM_0013018	Mis1:NM_03	0.003059	0.002379	0.01087
BOC	c.925G>A	p.Gly309Arg	NM_0013018	Mis2:NM_03	0.0004371	0	0.005435
BOC	c.964C>T	p.Pro322Ser	NM_0013018	Mis3:NM_03	0.0004374	0.0004762	0
BOC	c.1031G>A	p.Cys344Tyr	NM_0013018	Mis3:NM_03	0.001748	0.0004757	0.0163
BOC	c.1031G>A	p.Cys344Tyr	NM_0013018	Mis3:NM_03	0.001748	0.0004757	0.0163
BOC	c.1031G>A	p.Cys344Tyr	NM_0013018	Mis3:NM_03	0.001748	0.0004757	0.0163
BOC	c.1031G>A	p.Cys344Tyr	NM_0013018	Mis3:NM_03	0.001748	0.0004757	0.0163
BOC	c.1040G>A	p.Arg347His	NM_0013018	Mis1:NM_03	0.0004371	0.0004757	0
BOC	c.1108C>T	p.Arg370Trp	NM_0013018	Mis3:NM_03	0.0004371	0.0004757	0
BOC	c.1118G>A	p.Arg373His	NM_0013018	Mis1:NM_03	0.0008741	0.0009515	0
BOC	c.1118G>A	p.Arg373His	NM_0013018	Mis1:NM_03	0.0008741	0.0009515	0
BOC	c.1186G>C	p.Glu396Gln	NM_0013018	Mis1:NM_03	0.001311	0.0009515	0.005435
BOC	c.1186G>C	p.Glu396Gln	NM_0013018	Mis1:NM_03	0.001311	0.0009515	0.005435
BOC	c.1186G>C	p.Glu396Gln	NM_0013018	Mis1:NM_03	0.001311	0.0009515	0.005435
BOC	c.1505G>A	p.Arg502Gln	NM_0013018	Mis1:NM_03	0.0004382	0.0004766	0
BOC	c.1514T>C	p.Ile505Thr	NM_0013018	Mis3:NM_03	0.0004386	0.0004771	0
BOC	c.1667C>T	p.Ala556Val	NM_0013018	Mis2:NM_03	0.0004371	0.0004757	0
BOC	c.2074G>A	p.Glu692Lys	NM_0013018	Mis2:NM_03	0.0004371	0	0.005435
BOC	c.2233C>T	p.Pro745Ser	NM_0013018	Mis3:NM_03	0.0004371	0.0004757	0
BOC	c.2314G>T	p.Asp772Tyr	NM_0013018	Mis3:NM_03	0.0004371	0.0004757	0
BOC	c.2401G>A	p.Glu801Lys	NM_0013018	Mis3:NM_03	0.0004371	0.0004757	0
BOC	c.2573T>C	p.Ile858Thr	NM_0013018	Mis3:NM_03	0.0004371	0.0004757	0
BOC	c.2652G>C	p.Lys884Asn	NM_0013018	Mis2:NM_03	0.0004371	0.0004757	0
BOC	c.2701T>C	p.Ser901Pro	NM_0013018	Mis2:NM_03	0.0004371	0	0.005435
BOC	c.2785C>T	p.Arg929Trp	NM_0013018	Mis2:NM_03	0.0004371	0	0.005435
BOC	c.2786G>A	p.Arg929Gln	NM_0013018	Mis1:NM_03	0.0004371	0.0004757	0
BOC	c.2900C>T	p.Thr967Ile	NM_0013018	Mis1:NM_03	0.0004371	0.0004757	0

ExAC_ALL_AF	ExAC_NFE_AI	ExAC_SAS_AF	ExAC_AMR_A	ExAC_AFR_AI	ExAC_EAS_AF	ExAC_OTH_A	ExAC_FIN_AF
0.0130	0.0003	8.804e-05	0.0069	0.1318	0	0.0076	0
0.0001	4.63e-05	0	0.0004	0.0010	0	0	0
NA	NA	NA	NA	NA	NA	NA	NA
8.238e-06	0	0	0	0	0	0.0011	0
0.0006	0.0008	0.0005	0.0011	0.0002	0	0	0.0002
0.0006	0.0008	0.0005	0.0011	0.0002	0	0	0.0002
0.0006	0.0008	0.0005	0.0011	0.0002	0	0	0.0002
0.0006	0.0008	0.0005	0.0011	0.0002	0	0	0.0002
0.0006	0.0008	0.0005	0.0011	0.0002	0	0	0.0002
0.0006	0.0008	0.0005	0.0011	0.0002	0	0	0.0002
0.0006	0.0008	0.0005	0.0011	0.0002	0	0	0.0002
0.0005	0.0003	6.069e-05	0	9.66e-05	0.0039	0.0034	0
NA	NA	NA	NA	NA	NA	NA	NA
0.0007	0.0010	0.0002	0.0003	0.0005	0	0.0011	0
0.0007	0.0010	0.0002	0.0003	0.0005	0	0.0011	0
0.0007	0.0010	0.0002	0.0003	0.0005	0	0.0011	0
0.0007	0.0010	0.0002	0.0003	0.0005	0	0.0011	0
NA	NA	NA	NA	NA	NA	NA	NA
3.363e-05	4.595e-05	0	8.754e-05	0	0	0	0
4.233e-05	4.63e-05	0	0	0.0001	0.0001	0	0
4.233e-05	4.63e-05	0	0	0.0001	0.0001	0	0
0.0004	0.0007	0	0.0005	0.0004	0	0	0
0.0004	0.0007	0	0.0005	0.0004	0	0	0
0.0004	0.0007	0	0.0005	0.0004	0	0	0
0.0001	.	.	.	.	.	.	.
NA	NA	NA	NA	NA	NA	NA	NA
1.648e-05	2.998e-05	0	0	0	0	0	0
5.808e-05	6.021e-05	0	0.0003	0	0	0	0
NA	NA	NA	NA	NA	NA	NA	NA
NA	NA	NA	NA	NA	NA	NA	NA
8.243e-06	1.499e-05	0	0	0	0	0	0
NA	NA	NA	NA	NA	NA	NA	NA
0.0096	0.0002	6.101e-05	0.0053	0.1046	0	0.0055	0
NA	NA	NA	NA	NA	NA	NA	NA
2.473e-05	4.5e-05	0	0	0	0	0	0
NA	NA	NA	NA	NA	NA	NA	NA
NA	NA	NA	NA	NA	NA	NA	NA

ExAC_maxPO	SIFT	Polyphen2_H	Mutation_tas	pLI score	gTEX_Mean_	gTEX_mean_l	gTEX_mean_
0.1318	D,D	B	N	0.00472749	7.06	1.41	8.89
0.0010	T,D,T,T	P,P	D	0.00472749	7.06	1.41	8.89
NA	T,D,T,T	B,B	D	0.00472749	7.06	1.41	8.89
0.0011	T,T,T	P,P	D	0.00472749	7.06	1.41	8.89
0.0011	T,T,T	B,B	D	0.00472749	7.06	1.41	8.89
0.0011	T,T,T	B,B	D	0.00472749	7.06	1.41	8.89
0.0011	T,T,T	B,B	D	0.00472749	7.06	1.41	8.89
0.0011	T,T,T	B,B	D	0.00472749	7.06	1.41	8.89
0.0011	T,T,T	B,B	D	0.00472749	7.06	1.41	8.89
0.0011	T,T,T	B,B	D	0.00472749	7.06	1.41	8.89
0.0011	T,T,T	B,B	D	0.00472749	7.06	1.41	8.89
0.0039	T,T,T	D,D	D	0.00472749	7.06	1.41	8.89
NA	D,D,D	P,P	D	0.00472749	7.06	1.41	8.89
0.0011	D,D,D	D,D	D	0.00472749	7.06	1.41	8.89
0.0011	D,D,D	D,D	D	0.00472749	7.06	1.41	8.89
0.0011	D,D,D	D,D	D	0.00472749	7.06	1.41	8.89
0.0011	D,D,D	D,D	D	0.00472749	7.06	1.41	8.89
NA	T,T,T	B,B	D	0.00472749	7.06	1.41	8.89
8.754e-05	D,D,D	D,D	D	0.00472749	7.06	1.41	8.89
0.0001	T,T,T	B,B	D	0.00472749	7.06	1.41	8.89
0.0001	T,T,T	B,B	D	0.00472749	7.06	1.41	8.89
0.0007	T,T,T	B,B	D	0.00472749	7.06	1.41	8.89
0.0007	T,T,T	B,B	D	0.00472749	7.06	1.41	8.89
0.0007	T,T,T	B,B	D	0.00472749	7.06	1.41	8.89
0.0001	T,T,T	B,B	D	0.00472749	7.06	1.41	8.89
NA	D,D,D	P,P	D	0.00472749	7.06	1.41	8.89
2.998e-05	T,T,T	D,D	D	0.00472749	7.06	1.41	8.89
0.0003	T,T,T	P,P,B	D	0.00472749	7.06	1.41	8.89
NA	D,D,D	D,D,D	D	0.00472749	7.06	1.41	8.89
NA	D,D,D	D,D,D	D	0.00472749	7.06	1.41	8.89
1.499e-05	D,D,D	D,D,D	D	0.00472749	7.06	1.41	8.89
NA	D,D,D	D,P,P	D	0.00472749	7.06	1.41	8.89
0.1046	T,T,T	D,D,D	D	0.00472749	7.06	1.41	8.89
NA	D,D,D	B,B,B	D	0.00472749	7.06	1.41	8.89
4.5e-05	T,T,T	D,D,D	D	0.00472749	7.06	1.41	8.89
NA	T,T,T	B,B,B	D	0.00472749	7.06	1.41	8.89
NA	.,D,D,D	.,B,B,B	N,N	0.00472749	7.06	1.41	8.89

gTEX_Diff_Exp_brain/other
0.159
0.159
0.159
0.159
0.159
0.159
0.159
0.159
0.159
0.159
0.159
0.159
0.159
0.159
0.159
0.159
0.159
0.159
0.159
0.159
0.159
0.159
0.159
0.159
0.159
0.159
0.159
0.159
0.159
0.159
0.159
0.159
0.159
0.159
0.159
0.159
0.159

Sample	Status	Chr	Position	ID	Ref	Alt	Type
PMD-BOH-08	1	3	132047117	chr3_132047	C	T	SD
20662	0	3	132050522	chr3_132050	A	G	MD
B00GWBA	0	3	132050528	chr3_132050	T	C	BD
PMD-DUC-08	1	3	132056298	chr3_132056	A	G	LOF
PMD-FLA-082	1	3	132075555	chr3_132075	C	T	SD

Gene	BaseChange	AAChange	Transcript	OtherTranscr	MAF	MAFControls	MAFCases
ACPP	c.127C>T	p.Arg43Trp	NM_0011341	SD:NM_0010	0.0004371	0	0.005435
ACPP	c.248A>G	p.Glu83Gly	NM_0011341	MD:NM_001	0.0004421	0.0004817	0
ACPP	c.254T>C	p.Ile85Thr	NM_0011341	BD:NM_0010	0.0004421	0.0004817	0
ACPP	c.457-2A>G		NM_0011341	LOF:NM_001	0.0004371	0	0.005435
ACPP	c.994C>T	p.Arg332Trp	NM_0011341	SD:NM_0010	0.0004371	0	0.005435

ExAC_ALL_AF	ExAC_NFE_AI	ExAC_SAS_AF	ExAC_AMR_A	ExAC_AFR_AI	ExAC_EAS_AF	ExAC_OTH_A	ExAC_FIN_AF
8.271e-06	0	0	8.672e-05	0	0	0	0
0.0001	0.0001	0.0002	0.0002	0	0	0.0011	0
3.328e-05	6.038e-05	0	0	0	0	0	0
8.266e-06	1.502e-05	0	0	0	0	0	0
8.238e-06	1.499e-05	0	0	0	0	0	0

ExAC_maxPO	SIFT	Polyphen2_H	Mutation_tas	pLI score	gTEX_Mean_	gTEX_mean_	gTEX_mean_
8.672e-05	D,D,D,D	D,D,D	D	1.76E-07	12.05	0.05	15.95
0.0011	D,D,D	P,P,P	N	1.76E-07	12.05	0.05	15.95
6.038e-05	D,D,T	B,B,B	N	1.76E-07	12.05	0.05	15.95
1.502e-05			D	1.76E-07	12.05	0.05	15.95
1.499e-05	D,D,D	D,D,D	D	1.76E-07	12.05	0.05	15.95

gTEX_Diff_Exp_brain/other
0.003
0.003
0.003
0.003
0.003

Sample	Status	Chr	Position	ID	Ref	Alt	Type
22167	0	5	41313783	chr5_413137	C	T	Mis2
B00GG5P	0	5	41313840	chr5_413138	G	A	Mis2
PMis2-DEI-08	1	5	41313844	chr5_413138	G	A	Mis3
PMis2-FAL-08	1	5	41382576	chr5_413825	G	C	Mis3
PMis2-DAN-0	1	5	41382576	chr5_413825	G	C	Mis3

Gene	BaseChange	AAChange	Transcript	OtherTranscr	MAF	MAFControls	MAFCases
PLCXD3	c.902G>A	p.Gly301Asp	NM_001005473.2		0.0004371	0.0004757	0
PLCXD3	c.845C>T	p.Thr282Met	NM_001005473.2		0.0004371	0.0004757	0
PLCXD3	c.841C>T	p.Arg281Cys	NM_001005473.2		0.0004371	0	0.005435
PLCXD3	c.164C>G	p.Pro55Arg	NM_001005473.2		0.0008741	0	0.01087
PLCXD3	c.164C>G	p.Pro55Arg	NM_001005473.2		0.0008741	0	0.01087

ExAC_ALL_AF	ExAC_NFE_AI	ExAC_SAS_AF	ExAC_AMR_A	ExAC_AFR_AI	ExAC_EAS_AF	ExAC_OTH_A	ExAC_FIN_AF
0.0001	0.0001	0	0	0.0004	0	0.0011	0
0.0002	0.0003	0	0	0	0.0001	0	0
2.551e-05	0	0.0001	0	0	0.0001	0	0
5.941e-05	1.538e-05	0.0003	0	0	0	0.0011	0
5.941e-05	1.538e-05	0.0003	0	0	0	0.0011	0

ExAC_maxPO	SIFT	Polyphen2_H	Mutation_tas	pLI score	gTEX_Mean_	gTEX_mean_	gTEX_mean_
0.0011	T,T	D	D	0.04848874	2.75	4.08	2.32
0.0003	T,T	D	D	0.04848874	2.75	4.08	2.32
0.0001	D,D	D	D	0.04848874	2.75	4.08	2.32
0.0011	D,D	D	D	0.04848874	2.75	4.08	2.32
0.0011	D,D	D	D	0.04848874	2.75	4.08	2.32

gTEX_Diff_Exp_brain/other
1.759
1.759
1.759
1.759
1.759

Sample	Status	Chr	Position	ID	Ref	Alt	Type
PMD-NOT-09	1	5	60241171	chr5_602411	A	G	Mis3
B00GG7S	0	5	60448573	chr5_604485	A	G	Mis3
PMD-OUI-083	1	5	60448723	chr5_604487	G	A	Mis3
PMD-BUQ-08	1	5	60448723	chr5_604487	G	A	Mis3
PMD-VIA-070	1	5	60448723	chr5_604487	G	A	Mis3
B00FX00	0	5	60448723	chr5_604487	G	A	Mis3

Gene	BaseChange	AAChange	Transcript	OtherTranscr	MAF	MAFControls	MAFCases
NDUFAF2	c.89A>G	p.Asn30Ser	NM_174889.4		0.0004371	0	0.005435
NDUFAF2	c.301A>G	p.Lys101Glu	NM_174889.4		0.0004437	0.0004836	0
NDUFAF2	c.451G>A	p.Gly151Ser	NM_174889.4		0.001748	0.0004757	0.0163
NDUFAF2	c.451G>A	p.Gly151Ser	NM_174889.4		0.001748	0.0004757	0.0163
NDUFAF2	c.451G>A	p.Gly151Ser	NM_174889.4		0.001748	0.0004757	0.0163
NDUFAF2	c.451G>A	p.Gly151Ser	NM_174889.4		0.001748	0.0004757	0.0163

ExAC_ALL_AF	ExAC_NFE_A	ExAC_SAS_AF	ExAC_AMR_A	ExAC_AFR_A	ExAC_EAS_AF	ExAC_OTH_A	ExAC_FIN_AF
NA	NA	NA	NA	NA	NA	NA	NA
2.268e-05	4.818e-05	0	0	0	0	0	0
0.0053	0.0070	0.0007	0.0008	0.0003	0	0.0061	0.0215
0.0053	0.0070	0.0007	0.0008	0.0003	0	0.0061	0.0215
0.0053	0.0070	0.0007	0.0008	0.0003	0	0.0061	0.0215
0.0053	0.0070	0.0007	0.0008	0.0003	0	0.0061	0.0215

ExAC_maxPO	SIFT	Polyphen2_H	Mutation_tas	pLI score	gTEX_Mean_	gTEX_mean_	gTEX_mean_
NA	D,D,.	D,.	D,D	3.86E-07	11.7	10.65	12.04
4.818e-05	D	D	D	3.86E-07	11.7	10.65	12.04
0.0215	D	D	D	3.86E-07	11.7	10.65	12.04
0.0215	D	D	D	3.86E-07	11.7	10.65	12.04
0.0215	D	D	D	3.86E-07	11.7	10.65	12.04
0.0215	D	D	D	3.86E-07	11.7	10.65	12.04

gTEX_Diff_Exp_brain/other
0.884
0.884
0.884
0.884
0.884
0.884

Sample	Status	Chr	Position	ID	Ref	Alt	Type
PMD-BOC-08	1	6	33231323	chr6_332313	G	A	Mis3
B00GWBA	0	6	33234999	chr6_332349	A	G	Mis3
B00GG7C	0	6	33236290	chr6_332362	G	A	Mis3
21803	0	6	33236299	chr6_332362	C	T	Mis3
PMD-LEF-093	1	6	33236299	chr6_332362	C	T	Mis3
ROU-LEB-144	0	6	33236906	chr6_332369	G	A	Mis3
PMD-ROY-08	1	6	33236926	chr6_332369	C	G	Mis3
PMD-MAL-08	1	6	33236926	chr6_332369	C	G	Mis3
B00GG7F	0	6	33237527	chr6_332375	G	A	Mis3
PMD-HER-08	1	6	33237584	chr6_332375	G	A	Mis3

Gene	BaseChange	AAChange	Transcript	OtherTranscr	MAF	MAFControls	MAFCases
VPS52	c.1732C>T	p.Arg578Trp	NM_022553..	Mis3:NM_00	0.0004371	0	0.005435
VPS52	c.1091T>C	p.Leu364Pro	NM_022553..	Mis3:NM_00	0.0004371	0.0004757	0
VPS52	c.685C>T	p.Arg229Trp	NM_022553..	Mis3:NM_00	0.0004371	0.0004757	0
VPS52	c.676G>A	p.Val226Met	NM_022553..	Mis3:NM_00	0.0008741	0.0004757	0.005435
VPS52	c.676G>A	p.Val226Met	NM_022553..	Mis3:NM_00	0.0008741	0.0004757	0.005435
VPS52	c.433C>T	p.Arg145Trp	NM_022553..	Mis3:NM_00	0.0004371	0.0004757	0
VPS52	c.413G>C	p.Ser138Thr	NM_022553..	Mis3:NM_00	0.0008741	0	0.01087
VPS52	c.413G>C	p.Ser138Thr	NM_022553..	Mis3:NM_00	0.0008741	0	0.01087
VPS52	c.298C>T	p.Arg100Trp	NM_022553..	Mis3:NM_00	0.0004386	0.0004776	0
VPS52	c.241C>T	p.Arg81Cys	NM_022553..	Mis3:NM_00	0.0004371	0	0.005435

ExAC_ALL_AF	ExAC_NFE_A	ExAC_SAS_A	ExAC_AMR_A	ExAC_AFR_A	ExAC_EAS_A	ExAC_OTH_A	ExAC_FIN_AF
9.908e-05	0.0002	0	0	0.0002	0	0	0
NA	NA	NA	NA	NA	NA	NA	NA
1.775e-05	3.212e-05	0	0	0	0	0	0
3.503e-05	1.596e-05	6.678e-05	0	0	0.0002	0	0
3.503e-05	1.596e-05	6.678e-05	0	0	0.0002	0	0
3.439e-05	6.31e-05	0	0	0	0	0	0
6.021e-05	7.891e-05	0	0	0.0002	0	0	0
6.021e-05	7.891e-05	0	0	0.0002	0	0	0
8.32e-06	1.511e-05	0	0	0	0	0	0
7.468e-05	0.0001	0	8.735e-05	0	0	0	0

ExAC_maxPO	SIFT	Polyphen2_H	Mutation_tas	pLI score	gTEX_Mean_	gTEX_mean_	gTEX_mean_
0.0002	D,D,D	D,D	D	0.01596326	13.12	10.36	14.02
NA	D,D,D	D	D	0.01596326	13.12	10.36	14.02
3.212e-05	D,D,D	D,D,D	D	0.01596326	13.12	10.36	14.02
0.0002	D,D,D	D,P,D	D	0.01596326	13.12	10.36	14.02
0.0002	D,D,D	D,P,D	D	0.01596326	13.12	10.36	14.02
6.31e-05	D,D,D	D,D,D	D	0.01596326	13.12	10.36	14.02
0.0002	D,D,D	P,P,P	D	0.01596326	13.12	10.36	14.02
0.0002	D,D,D	P,P,P	D	0.01596326	13.12	10.36	14.02
1.511e-05	D,D	D,D	D	0.01596326	13.12	10.36	14.02
0.0001	D,D	D,D	D	0.01596326	13.12	10.36	14.02

gTEX_Diff_Exp_brain/other
0.739
0.739
0.739
0.739
0.739
0.739
0.739
0.739
0.739
0.739

Sample	Status	Chr	Position	ID	Ref	Alt	Type
ROU-REN-472	0	6	43395717	chr6_433957	A	G	PTV
MET01-225	1	6	43395717	chr6_433957	A	G	PTV
B00FWWM	0	6	43395717	chr6_433957	A	G	PTV
21352	0	6	43395733	chr6_433957	C	T	Mis1
ROU-DUH-43	0	6	43399957	chr6_433999	C	T	Mis1
21483	0	6	43400205	chr6_434002	C	G	Mis3
B00G74W	0	6	43400205	chr6_434002	C	G	Mis3
B00GQCF	0	6	43400205	chr6_434002	C	G	Mis3
PMis2-ETA-09	1	6	43400205	chr6_434002	C	G	Mis3
21727	0	6	43400205	chr6_434002	C	G	Mis3
B00GQBK	0	6	43400205	chr6_434002	C	G	Mis3
B00G7LO	0	6	43400205	chr6_434002	C	G	Mis3
PMis2-LAF-07	1	6	43400442	chr6_434004	C	T	Mis2
B00G74G	0	6	43400500	chr6_434005	G	A	Mis1
PMis2-HER-08	1	6	43400500	chr6_434005	G	A	Mis1
B00G72R	0	6	43400500	chr6_434005	G	A	Mis1
23247	0	6	43400500	chr6_434005	G	A	Mis1
21463	0	6	43400500	chr6_434005	G	A	Mis1
B00FWXM	0	6	43400500	chr6_434005	G	A	Mis1
B00G73U	0	6	43400500	chr6_434005	G	A	Mis1
B00G7LP	0	6	43400500	chr6_434005	G	A	Mis1
PMis2-LEC-08	1	6	43400500	chr6_434005	G	A	Mis1
23041	0	6	43400500	chr6_434005	G	A	Mis1
ROU-PAI-560	0	6	43400500	chr6_434005	G	A	Mis1
B00G7KX	0	6	43400500	chr6_434005	G	A	Mis1
ROU-JOL-330	0	6	43400500	chr6_434005	G	A	Mis1
PMis2-LOG-0	1	6	43400500	chr6_434005	G	A	Mis1
21839	0	6	43400500	chr6_434005	G	A	Mis1
B00FWX2	0	6	43400500	chr6_434005	G	A	Mis1
B00GG7C	0	6	43400785	chr6_434007	G	A	Mis3
B00GG80	0	6	43400839	chr6_434008	G	A	Mis3
B00GQDW	0	6	43400841	chr6_434008	C	T	Mis1
B00G7LO	0	6	43400850	chr6_434008	G	A	Mis3
B00FWXV	0	6	43401027	chr6_434010	A	G	Mis3
B00GG7U	0	6	43401027	chr6_434010	A	G	Mis3
20720	0	6	43401027	chr6_434010	A	G	Mis3
PMis2-MAL-0	1	6	43401027	chr6_434010	A	G	Mis3
20180	0	6	43401027	chr6_434010	A	G	Mis3
21291	0	6	43401027	chr6_434010	A	G	Mis3
20197	0	6	43401027	chr6_434010	A	G	Mis3
B00GG7F	0	6	43402567	chr6_434025	A	G	Mis3
22004	0	6	43403556	chr6_434035	A	G	Mis3
B00GWAJ	0	6	43403556	chr6_434035	A	G	Mis3
B00GWBS	0	6	43403556	chr6_434035	A	G	Mis3
PMis2-SCH-09	1	6	43406448	chr6_434064	G	A	Mis3
24688	0	6	43406475	chr6_434064	T	C	Mis3
20480	0	6	43406522	chr6_434065	G	T	Mis3
PMis2-DEL-08	1	6	43409667	chr6_434096	C	T	Mis3
PMis2-GAI-09	1	6	43410879	chr6_434108	G	A	Mis3

B00G72T	0	6	43410891	chr6_434108	C	T	Mis2
B00GQD0	0	6	43412080	chr6_434120	T	C	Mis3
PMis2-VIA-07	1	6	43413026	chr6_434130	C	T	Mis1
21221	0	6	43413041	chr6_434130	C	T	PTV
B00GG7M	0	6	43413442	chr6_434134	C	T	Mis1
B00FWYE	0	6	43413442	chr6_434134	C	T	Mis1
MET01-058	1	6	43413550	chr6_434135	C	T	PTV
B00GWBR	0	6	43413643	chr6_434136	G	C	Mis3
20352	0	6	43413652	chr6_434136	G	A	Mis3
22356	0	6	43413652	chr6_434136	G	A	Mis3
ROU-ETI-678-	0	6	43413652	chr6_434136	G	A	Mis3
B00G7L7	0	6	43413652	chr6_434136	G	A	Mis3
ROU-PAN-60:	0	6	43413652	chr6_434136	G	A	Mis3
ROU-FAU-143	0	6	43413652	chr6_434136	G	A	Mis3
22044	0	6	43413652	chr6_434136	G	A	Mis3
21128	0	6	43413652	chr6_434136	G	A	Mis3
21915	0	6	43413652	chr6_434136	G	A	Mis3
B00GQF4	0	6	43413652	chr6_434136	G	A	Mis3
21794	0	6	43413652	chr6_434136	G	A	Mis3
PMis2-FAM-0	1	6	43413652	chr6_434136	G	A	Mis3
21911	0	6	43413652	chr6_434136	G	A	Mis3
B00GG5L	0	6	43413658	chr6_434136	C	T	Mis3
B00G73K	0	6	43413658	chr6_434136	C	T	Mis3
B00G7LP	0	6	43415084	chr6_434150	G	A	Mis3
ROU-LEB-171	0	6	43415578	chr6_434155	T	C	Mis3
ROU-CLA-516	0	6	43415672	chr6_434156	T	C	Mis3
21407	0	6	43416639	chr6_434166	C	T	Mis3
20148	0	6	43416765	chr6_434167	G	C	PTV
PMis2-VIG-08	1	6	43416869	chr6_434168	A	C	Mis3
B00G720	0	6	43416877	chr6_434168	C	T	Mis2
PMis2-LEN-09	1	6	43417746	chr6_434177	C	A	Mis3
B00FWX7	0	6	43417795	chr6_434177	A	G	Mis1

Gene	BaseChange	AAChange	Transcript	OtherTranscr	MAF	MAFControls	MAFCases
ABCC10	c.1A>G	p.Met1?	NM_001198934.1		0.001311	0.0009515	0.005435
ABCC10	c.1A>G	p.Met1?	NM_001198934.1		0.001311	0.0009515	0.005435
ABCC10	c.1A>G	p.Met1?	NM_001198934.1		0.001311	0.0009515	0.005435
ABCC10	c.17C>T	p.Ala6Val	NM_001198934.1		0.0004371	0.0004757	0
ABCC10	c.239C>T	p.Pro80Leu	NM_0011989	Mis1:NM_03	0.0004371	0.0004757	0
ABCC10	c.487C>G	p.Pro163Ala	NM_0011989	Mis3:NM_03	0.003059	0.002854	0.005435
ABCC10	c.487C>G	p.Pro163Ala	NM_0011989	Mis3:NM_03	0.003059	0.002854	0.005435
ABCC10	c.487C>G	p.Pro163Ala	NM_0011989	Mis3:NM_03	0.003059	0.002854	0.005435
ABCC10	c.487C>G	p.Pro163Ala	NM_0011989	Mis3:NM_03	0.003059	0.002854	0.005435
ABCC10	c.487C>G	p.Pro163Ala	NM_0011989	Mis3:NM_03	0.003059	0.002854	0.005435
ABCC10	c.487C>G	p.Pro163Ala	NM_0011989	Mis3:NM_03	0.003059	0.002854	0.005435
ABCC10	c.487C>G	p.Pro163Ala	NM_0011989	Mis3:NM_03	0.003059	0.002854	0.005435
ABCC10	c.724C>T	p.Arg242Trp	NM_0011989	Mis2:NM_03	0.0004371	0	0.005435
ABCC10	c.782G>A	p.Arg261His	NM_0011989	Mis1:NM_03	0.006993	0.006185	0.0163
ABCC10	c.782G>A	p.Arg261His	NM_0011989	Mis1:NM_03	0.006993	0.006185	0.0163
ABCC10	c.782G>A	p.Arg261His	NM_0011989	Mis1:NM_03	0.006993	0.006185	0.0163
ABCC10	c.782G>A	p.Arg261His	NM_0011989	Mis1:NM_03	0.006993	0.006185	0.0163
ABCC10	c.782G>A	p.Arg261His	NM_0011989	Mis1:NM_03	0.006993	0.006185	0.0163
ABCC10	c.782G>A	p.Arg261His	NM_0011989	Mis1:NM_03	0.006993	0.006185	0.0163
ABCC10	c.782G>A	p.Arg261His	NM_0011989	Mis1:NM_03	0.006993	0.006185	0.0163
ABCC10	c.782G>A	p.Arg261His	NM_0011989	Mis1:NM_03	0.006993	0.006185	0.0163
ABCC10	c.782G>A	p.Arg261His	NM_0011989	Mis1:NM_03	0.006993	0.006185	0.0163
ABCC10	c.782G>A	p.Arg261His	NM_0011989	Mis1:NM_03	0.006993	0.006185	0.0163
ABCC10	c.782G>A	p.Arg261His	NM_0011989	Mis1:NM_03	0.006993	0.006185	0.0163
ABCC10	c.782G>A	p.Arg261His	NM_0011989	Mis1:NM_03	0.006993	0.006185	0.0163
ABCC10	c.782G>A	p.Arg261His	NM_0011989	Mis1:NM_03	0.006993	0.006185	0.0163
ABCC10	c.782G>A	p.Arg261His	NM_0011989	Mis1:NM_03	0.006993	0.006185	0.0163
ABCC10	c.782G>A	p.Arg261His	NM_0011989	Mis1:NM_03	0.006993	0.006185	0.0163
ABCC10	c.782G>A	p.Arg261His	NM_0011989	Mis1:NM_03	0.006993	0.006185	0.0163
ABCC10	c.1067G>A	p.Arg356Gln	NM_0011989	Mis3:NM_03	0.0004371	0.0004757	0
ABCC10	c.1121G>A	p.Arg374His	NM_0011989	Mis3:NM_03	0.0004371	0.0004757	0
ABCC10	c.1123C>T	p.Pro375Ser	NM_0011989	Mis1:NM_03	0.0004371	0.0004757	0
ABCC10	c.1132G>A	p.Gly378Arg	NM_0011989	Mis3:NM_03	0.0004371	0.0004757	0
ABCC10	c.1309A>G	p.Asn437Asp	NM_0011989	Mis3:NM_03	0.003059	0.002854	0.005435
ABCC10	c.1309A>G	p.Asn437Asp	NM_0011989	Mis3:NM_03	0.003059	0.002854	0.005435
ABCC10	c.1309A>G	p.Asn437Asp	NM_0011989	Mis3:NM_03	0.003059	0.002854	0.005435
ABCC10	c.1309A>G	p.Asn437Asp	NM_0011989	Mis3:NM_03	0.003059	0.002854	0.005435
ABCC10	c.1309A>G	p.Asn437Asp	NM_0011989	Mis3:NM_03	0.003059	0.002854	0.005435
ABCC10	c.1309A>G	p.Asn437Asp	NM_0011989	Mis3:NM_03	0.003059	0.002854	0.005435
ABCC10	c.1309A>G	p.Asn437Asp	NM_0011989	Mis3:NM_03	0.003059	0.002854	0.005435
ABCC10	c.1589A>G	p.His530Arg	NM_0011989	Mis3:NM_03	0.0004371	0.0004757	0
ABCC10	c.1676A>G	p.Asn559Ser	NM_0011989	Mis3:NM_03	0.001311	0.001427	0
ABCC10	c.1676A>G	p.Asn559Ser	NM_0011989	Mis3:NM_03	0.001311	0.001427	0
ABCC10	c.1676A>G	p.Asn559Ser	NM_0011989	Mis3:NM_03	0.001311	0.001427	0
ABCC10	c.2042G>A	p.Arg681Gln	NM_0011989	Mis3:NM_03	0.0004371	0	0.005435
ABCC10	c.2069T>C	p.Phe690Ser	NM_0011989	Mis3:NM_03	0.0004371	0.0004757	0
ABCC10	c.2116G>T	p.Asp706Tyr	NM_0011989	Mis3:NM_03	0.0004371	0.0004757	0
ABCC10	c.2195C>T	p.Ala732Val	NM_0011989	Mis3:NM_03	0.0004371	0	0.005435
ABCC10	c.2398G>A	p.Gly800Arg	NM_0011989	Mis3:NM_03	0.0004452	0	0.005495

ABCC10	c.2410C>T	p.Arg804Trp	NM_0011989	Mis2:NM_03	0.0004517	0.0004921	0
ABCC10	c.2678T>C	p.Leu893Pro	NM_0011989	Mis3:NM_03	0.0004378	0.0004766	0
ABCC10	c.3004C>T	p.Arg1002Cys	NM_0011989	Mis1:NM_03	0.0004371	0	0.005435
ABCC10	c.3019C>T	p.Arg1007*	NM_0011989	PTV:NM_033	0.0004371	0.0004757	0
ABCC10	c.3136C>T	p.Leu1046Ph	NM_0011989	Mis1:NM_03	0.0008741	0.0009515	0
ABCC10	c.3136C>T	p.Leu1046Ph	NM_0011989	Mis1:NM_03	0.0008741	0.0009515	0
ABCC10	c.3244C>T	p.Gln1082*	NM_0011989	PTV:NM_033	0.0004371	0	0.005435
ABCC10	c.3337G>C	p.Gly1113Arg	NM_0011989	Mis3:NM_03	0.0004371	0.0004757	0
ABCC10	c.3346G>A	p.Val1116Me	NM_0011989	Mis3:NM_03	0.005682	0.005709	0.005435
ABCC10	c.3346G>A	p.Val1116Me	NM_0011989	Mis3:NM_03	0.005682	0.005709	0.005435
ABCC10	c.3346G>A	p.Val1116Me	NM_0011989	Mis3:NM_03	0.005682	0.005709	0.005435
ABCC10	c.3346G>A	p.Val1116Me	NM_0011989	Mis3:NM_03	0.005682	0.005709	0.005435
ABCC10	c.3346G>A	p.Val1116Me	NM_0011989	Mis3:NM_03	0.005682	0.005709	0.005435
ABCC10	c.3346G>A	p.Val1116Me	NM_0011989	Mis3:NM_03	0.005682	0.005709	0.005435
ABCC10	c.3346G>A	p.Val1116Me	NM_0011989	Mis3:NM_03	0.005682	0.005709	0.005435
ABCC10	c.3346G>A	p.Val1116Me	NM_0011989	Mis3:NM_03	0.005682	0.005709	0.005435
ABCC10	c.3346G>A	p.Val1116Me	NM_0011989	Mis3:NM_03	0.005682	0.005709	0.005435
ABCC10	c.3346G>A	p.Val1116Me	NM_0011989	Mis3:NM_03	0.005682	0.005709	0.005435
ABCC10	c.3346G>A	p.Val1116Me	NM_0011989	Mis3:NM_03	0.005682	0.005709	0.005435
ABCC10	c.3346G>A	p.Val1116Me	NM_0011989	Mis3:NM_03	0.005682	0.005709	0.005435
ABCC10	c.3346G>A	p.Val1116Me	NM_0011989	Mis3:NM_03	0.005682	0.005709	0.005435
ABCC10	c.3352C>T	p.Arg1118Trp	NM_0011989	Mis3:NM_03	0.0008741	0.0009515	0
ABCC10	c.3352C>T	p.Arg1118Trp	NM_0011989	Mis3:NM_03	0.0008741	0.0009515	0
ABCC10	c.3643G>A	p.Val1215Ile	NM_0011989	Mis3:NM_03	0.0004371	0.0004757	0
ABCC10	c.3862T>C	p.Ser1288Pro	NM_0011989	Mis3:NM_03	0.0004371	0.0004757	0
ABCC10	c.3956T>C	p.Leu1319Pro	NM_0011989	Mis3:NM_03	0.0004371	0.0004757	0
ABCC10	c.3980C>T	p.Pro1327Leu	NM_0011989	Mis3:NM_03	0.0004371	0.0004757	0
ABCC10	c.4105+1G>C		NM_0011989	PTV:NM_033	0.0004371	0.0004757	0
ABCC10	c.4130A>C	p.Glu1377Ala	NM_0011989	Mis3:NM_03	0.0004371	0	0.005435
ABCC10	c.4138C>T	p.Arg1380Trp	NM_0011989	Mis2:NM_03	0.0004371	0.0004757	0
ABCC10	c.4396C>A	p.Leu1466Me	NM_0011989	Mis3:NM_03	0.0004378	0	0.005435
ABCC10	c.4445A>G	p.Gln1482Arg	NM_0011989	Mis1:NM_03	0.0004413	0.0004798	0

ExAC_ALL_AF	ExAC_NFE_AI	ExAC_SAS_AF	ExAC_AMR_A	ExAC_AFR_AI	ExAC_EAS_AF	ExAC_OTH_A	ExAC_FIN_AF
0.0003	0.0005	0	0.0004	0	0	0	0.0002
0.0003	0.0005	0	0.0004	0	0	0	0.0002
0.0003	0.0005	0	0.0004	0	0	0	0.0002
NA	NA	NA	NA	NA	NA	NA	NA
1.648e-05	1.499e-05	6.056e-05	0	0	0	0	0
0.0010	0.0016	6.056e-05	0.0012	0.0002	0	0	0.0003
0.0010	0.0016	6.056e-05	0.0012	0.0002	0	0	0.0003
0.0010	0.0016	6.056e-05	0.0012	0.0002	0	0	0.0003
0.0010	0.0016	6.056e-05	0.0012	0.0002	0	0	0.0003
0.0010	0.0016	6.056e-05	0.0012	0.0002	0	0	0.0003
0.0010	0.0016	6.056e-05	0.0012	0.0002	0	0	0.0003
0.0010	0.0016	6.056e-05	0.0012	0.0002	0	0	0.0003
1.674e-05	3.058e-05	0	0	0	0	0	0
0.0035	0.0054	6.058e-05	0.0035	0.0012	0	0.0044	0.0003
0.0035	0.0054	6.058e-05	0.0035	0.0012	0	0.0044	0.0003
0.0035	0.0054	6.058e-05	0.0035	0.0012	0	0.0044	0.0003
0.0035	0.0054	6.058e-05	0.0035	0.0012	0	0.0044	0.0003
0.0035	0.0054	6.058e-05	0.0035	0.0012	0	0.0044	0.0003
0.0035	0.0054	6.058e-05	0.0035	0.0012	0	0.0044	0.0003
0.0035	0.0054	6.058e-05	0.0035	0.0012	0	0.0044	0.0003
0.0035	0.0054	6.058e-05	0.0035	0.0012	0	0.0044	0.0003
0.0035	0.0054	6.058e-05	0.0035	0.0012	0	0.0044	0.0003
0.0035	0.0054	6.058e-05	0.0035	0.0012	0	0.0044	0.0003
0.0035	0.0054	6.058e-05	0.0035	0.0012	0	0.0044	0.0003
0.0035	0.0054	6.058e-05	0.0035	0.0012	0	0.0044	0.0003
0.0035	0.0054	6.058e-05	0.0035	0.0012	0	0.0044	0.0003
0.0035	0.0054	6.058e-05	0.0035	0.0012	0	0.0044	0.0003
0.0035	0.0054	6.058e-05	0.0035	0.0012	0	0.0044	0.0003
0.0035	0.0054	6.058e-05	0.0035	0.0012	0	0.0044	0.0003
2.473e-05	3.00E-05	0	0	9.617e-05	0	0	0
7.416e-05	0.0001	0.0001	0	0	0	0	0
0.0001	0.0002	0	0	0	0	0	0
0.0002	0.0003	0	0.0009	0.0002	0	0	0
0.0034	0.0052	0.0002	0.0027	0.0010	0.0001	0.0022	0.0032
0.0034	0.0052	0.0002	0.0027	0.0010	0.0001	0.0022	0.0032
0.0034	0.0052	0.0002	0.0027	0.0010	0.0001	0.0022	0.0032
0.0034	0.0052	0.0002	0.0027	0.0010	0.0001	0.0022	0.0032
0.0034	0.0052	0.0002	0.0027	0.0010	0.0001	0.0022	0.0032
0.0034	0.0052	0.0002	0.0027	0.0010	0.0001	0.0022	0.0032
0.0034	0.0052	0.0002	0.0027	0.0010	0.0001	0.0022	0.0032
NA	NA	NA	NA	NA	NA	NA	NA
7.414e-05	1.499e-05	0.0002	0	0.0004	0.0001	0	0
7.414e-05	1.499e-05	0.0002	0	0.0004	0.0001	0	0
7.414e-05	1.499e-05	0.0002	0	0.0004	0.0001	0	0
8.237e-06	0	0	0	0	0.0001	0	0
0.0004	0.0006	0	8.637e-05	0.0003	0	0	0
0.0002	7.501e-05	0.0010	0	0	0	0	0
NA	NA	NA	NA	NA	NA	NA	NA
2.632e-05	1.575e-05	0.0001	0	0	0	0	0

0.0006	0.0003	0.0036	0	0	0	0	0.0002
NA	NA	NA	NA	NA	NA	NA	NA
0.0002	0.0002	0	0	0.0001	0	0	0.0005
9.402e-06	1.689e-05	0	0	0	0	0	0
0.0009	0.0014	0.0005	0	0.0002	0	0.0011	0.0006
0.0009	0.0014	0.0005	0	0.0002	0	0.0011	0.0006
NA	NA	NA	NA	NA	NA	NA	NA
NA	NA	NA	NA	NA	NA	NA	NA
0.0030	0.0047	0.0009	0.0014	0.0006	0	0.0023	0.0014
0.0030	0.0047	0.0009	0.0014	0.0006	0	0.0023	0.0014
0.0030	0.0047	0.0009	0.0014	0.0006	0	0.0023	0.0014
0.0030	0.0047	0.0009	0.0014	0.0006	0	0.0023	0.0014
0.0030	0.0047	0.0009	0.0014	0.0006	0	0.0023	0.0014
0.0030	0.0047	0.0009	0.0014	0.0006	0	0.0023	0.0014
0.0030	0.0047	0.0009	0.0014	0.0006	0	0.0023	0.0014
0.0030	0.0047	0.0009	0.0014	0.0006	0	0.0023	0.0014
0.0030	0.0047	0.0009	0.0014	0.0006	0	0.0023	0.0014
0.0030	0.0047	0.0009	0.0014	0.0006	0	0.0023	0.0014
0.0030	0.0047	0.0009	0.0014	0.0006	0	0.0023	0.0014
0.0030	0.0047	0.0009	0.0014	0.0006	0	0.0023	0.0014
0.0030	0.0047	0.0009	0.0014	0.0006	0	0.0023	0.0014
6.923e-05	7.863e-05	0	8.72e-05	0	0.0002	0	0
6.923e-05	7.863e-05	0	8.72e-05	0	0.0002	0	0
1.652e-05	0	6.056e-05	8.651e-05	0	0	0	0
NA	NA	NA	NA	NA	NA	NA	NA
NA	NA	NA	NA	NA	NA	NA	NA
NA	NA	NA	NA	NA	NA	NA	NA
NA	NA	NA	NA	NA	NA	NA	NA
NA	NA	NA	NA	NA	NA	NA	NA
0.0001	0.0002	6.068e-05	0	9.61e-05	0	0	0.0002
NA	NA	NA	NA	NA	NA	NA	NA
1.699e-05	3.053e-05	0	0	0	0	0	0

ExAC_maxPO	SIFT	Polyphen2_H	Mutation_tas	pLI score	gTEX_Mean_	gTEX_mean_l	gTEX_mean_
0.0005	D	B	D	8.31E-21	4.71	4.11	4.9
0.0005	D	B	D	8.31E-21	4.71	4.11	4.9
0.0005	D	B	D	8.31E-21	4.71	4.11	4.9
NA	T	B	D	8.31E-21	4.71	4.11	4.9
6.056e-05	T,T	B,B	D	8.31E-21	4.71	4.11	4.9
0.0016	D,D	P,P	D	8.31E-21	4.71	4.11	4.9
0.0016	D,D	P,P	D	8.31E-21	4.71	4.11	4.9
0.0016	D,D	P,P	D	8.31E-21	4.71	4.11	4.9
0.0016	D,D	P,P	D	8.31E-21	4.71	4.11	4.9
0.0016	D,D	P,P	D	8.31E-21	4.71	4.11	4.9
0.0016	D,D	P,P	D	8.31E-21	4.71	4.11	4.9
0.0016	D,D	P,P	D	8.31E-21	4.71	4.11	4.9
3.058e-05	D,D	D,D	N	8.31E-21	4.71	4.11	4.9
0.0054	T,T	B,P	N	8.31E-21	4.71	4.11	4.9
0.0054	T,T	B,P	N	8.31E-21	4.71	4.11	4.9
0.0054	T,T	B,P	N	8.31E-21	4.71	4.11	4.9
0.0054	T,T	B,P	N	8.31E-21	4.71	4.11	4.9
0.0054	T,T	B,P	N	8.31E-21	4.71	4.11	4.9
0.0054	T,T	B,P	N	8.31E-21	4.71	4.11	4.9
0.0054	T,T	B,P	N	8.31E-21	4.71	4.11	4.9
0.0054	T,T	B,P	N	8.31E-21	4.71	4.11	4.9
0.0054	T,T	B,P	N	8.31E-21	4.71	4.11	4.9
0.0054	T,T	B,P	N	8.31E-21	4.71	4.11	4.9
0.0054	T,T	B,P	N	8.31E-21	4.71	4.11	4.9
0.0054	T,T	B,P	N	8.31E-21	4.71	4.11	4.9
0.0054	T,T	B,P	N	8.31E-21	4.71	4.11	4.9
0.0054	T,T	B,P	N	8.31E-21	4.71	4.11	4.9
0.0054	T,T	B,P	N	8.31E-21	4.71	4.11	4.9
0.0054	T,T	B,P	N	8.31E-21	4.71	4.11	4.9
9.617e-05	D,D	D,D	D	8.31E-21	4.71	4.11	4.9
0.0001	D,D	P,D	D	8.31E-21	4.71	4.11	4.9
0.0002	T,T	B,B	D	8.31E-21	4.71	4.11	4.9
0.0009	D,D	D,D	D	8.31E-21	4.71	4.11	4.9
0.0052	D,D	D,D	D	8.31E-21	4.71	4.11	4.9
0.0052	D,D	D,D	D	8.31E-21	4.71	4.11	4.9
0.0052	D,D	D,D	D	8.31E-21	4.71	4.11	4.9
0.0052	D,D	D,D	D	8.31E-21	4.71	4.11	4.9
0.0052	D,D	D,D	D	8.31E-21	4.71	4.11	4.9
0.0052	D,D	D,D	D	8.31E-21	4.71	4.11	4.9
0.0052	D,D	D,D	D	8.31E-21	4.71	4.11	4.9
NA	D,D,D	B,D	D	8.31E-21	4.71	4.11	4.9
0.0004	D,D,D	D,B	D	8.31E-21	4.71	4.11	4.9
0.0004	D,D,D	D,B	D	8.31E-21	4.71	4.11	4.9
0.0004	D,D,D	D,B	D	8.31E-21	4.71	4.11	4.9
0.0001	D,D,D	D,D	D	8.31E-21	4.71	4.11	4.9
0.0006	D,D,D	D,D	D	8.31E-21	4.71	4.11	4.9
0.0010	T,D,D	B,P	D	8.31E-21	4.71	4.11	4.9
NA	D,D	D,D	D	8.31E-21	4.71	4.11	4.9
0.0001	D,D	D,D	D	8.31E-21	4.71	4.11	4.9

0.0036	D,D	B,D	N	8.31E-21	4.71	4.11	4.9
NA	D,D	D,D	D	8.31E-21	4.71	4.11	4.9
0.0005	D,D	B,B	N	8.31E-21	4.71	4.11	4.9
1.689e-05			A	8.31E-21	4.71	4.11	4.9
0.0014	T,T	B,B	D	8.31E-21	4.71	4.11	4.9
0.0014	T,T	B,B	D	8.31E-21	4.71	4.11	4.9
NA			A	8.31E-21	4.71	4.11	4.9
NA	D,D	D,D	D	8.31E-21	4.71	4.11	4.9
0.0047	D,D	P,P	D	8.31E-21	4.71	4.11	4.9
0.0047	D,D	P,P	D	8.31E-21	4.71	4.11	4.9
0.0047	D,D	P,P	D	8.31E-21	4.71	4.11	4.9
0.0047	D,D	P,P	D	8.31E-21	4.71	4.11	4.9
0.0047	D,D	P,P	D	8.31E-21	4.71	4.11	4.9
0.0047	D,D	P,P	D	8.31E-21	4.71	4.11	4.9
0.0047	D,D	P,P	D	8.31E-21	4.71	4.11	4.9
0.0047	D,D	P,P	D	8.31E-21	4.71	4.11	4.9
0.0047	D,D	P,P	D	8.31E-21	4.71	4.11	4.9
0.0047	D,D	P,P	D	8.31E-21	4.71	4.11	4.9
0.0047	D,D	P,P	D	8.31E-21	4.71	4.11	4.9
0.0047	D,D	P,P	D	8.31E-21	4.71	4.11	4.9
0.0047	D,D	P,P	D	8.31E-21	4.71	4.11	4.9
0.0002	D,D	D,D	D	8.31E-21	4.71	4.11	4.9
0.0002	D,D	D,D	D	8.31E-21	4.71	4.11	4.9
8.651e-05	D,D	D,P	D	8.31E-21	4.71	4.11	4.9
NA	D,D,D	D,D	D	8.31E-21	4.71	4.11	4.9
NA	D,D,D	D,D	D	8.31E-21	4.71	4.11	4.9
NA	D,D,D	D,D	D	8.31E-21	4.71	4.11	4.9
NA			D	8.31E-21	4.71	4.11	4.9
NA	D,D,D	P,P	D	8.31E-21	4.71	4.11	4.9
0.0002	D,D,D	D,D	N	8.31E-21	4.71	4.11	4.9
NA	D,D,D,D	D,D	D	8.31E-21	4.71	4.11	4.9
3.053e-05	T,T,T,T	B,B	D	8.31E-21	4.71	4.11	4.9

gTEX_Diff_Exp_brain/other
0.84
0.84
0.84
0.84
0.84
0.84
0.84
0.84
0.84
0.84
0.84
0.84
0.84
0.84
0.84
0.84
0.84
0.84
0.84
0.84
0.84
0.84
0.84
0.84
0.84
0.84
0.84
0.84
0.84
0.84
0.84
0.84
0.84
0.84
0.84
0.84
0.84
0.84
0.84
0.84
0.84
0.84
0.84
0.84
0.84
0.84
0.84
0.84
0.84

0.84
0.84
0.84
0.84
0.84
0.84
0.84
0.84
0.84
0.84
0.84
0.84
0.84
0.84
0.84
0.84
0.84
0.84
0.84
0.84
0.84
0.84
0.84
0.84
0.84
0.84
0.84
0.84
0.84
0.84
0.84
0.84

Sample	Status	Chr	Position	ID	Ref	Alt	Type
22445	0	9	15623326	chr9_156233	T	G	Mis1
ROU-TRA-481	0	9	15623346	chr9_156233	C	T	Mis2
PMis2-BEL-09	1	9	15874585	chr9_158745	T	G	Mis3
24640	0	9	15666309	chr9_156663	C	G	Mis1
20225	0	9	15695278	chr9_156952	G	A	Mis1
B00G72A	0	9	15695278	chr9_156952	G	A	Mis1
PMis2-BOU-0	1	9	15874585	chr9_158745	T	G	Mis3
21666	0	9	15721841	chr9_157218	T	C	Mis2
B00GG7A	0	9	15724872	chr9_157248	A	C	Mis2
PMis2-DEI-08	1	9	15971726	chr9_159717	C	G	Mis1
20713	0	9	15744297	chr9_157442	C	A	Mis2
PMis2-FOL-09	1	9	15727873	chr9_157278	C	G	Mis2
PMis2-GRO-0	1	9	15623346	chr9_156233	C	T	Mis2
PMis2-LEB-09	1	9	15874585	chr9_158745	T	G	Mis3
ROU-GED-415	0	9	15744541	chr9_157445	C	G	Mis2
B00G727	0	9	15745542	chr9_157455	C	G	Mis1
B00G74K	0	9	15745542	chr9_157455	C	G	Mis1
B00FWWL	0	9	15745596	chr9_157455	A	G	Mis1
21478	0	9	15777618	chr9_157776	G	A	Mis2
22167	0	9	15784616	chr9_157846	A	T	Mis3
B00G72B	0	9	15846815	chr9_158468	A	G	Mis3
22004	0	9	15874585	chr9_158745	T	G	Mis3
PMis2-LEL-09	1	9	15744298	chr9_157442	A	G	Mis1
20376	0	9	15874585	chr9_158745	T	G	Mis3
PMis2-LEV-07	1	9	15721827	chr9_157218	G	T	Mis1
22519	0	9	15874585	chr9_158745	T	G	Mis3
B00GG8P	0	9	15874585	chr9_158745	T	G	Mis3
B00G7JY	0	9	15874585	chr9_158745	T	G	Mis3
22712	0	9	15874585	chr9_158745	T	G	Mis3
B00FWXD	0	9	15874585	chr9_158745	T	G	Mis3
PMis2-LEV-07	1	9	15874585	chr9_158745	T	G	Mis3
PMis2-LOG-0	1	9	15744330	chr9_157443	TG	T	PTV
20705	0	9	15874585	chr9_158745	T	G	Mis3
B00GQF3	0	9	15874585	chr9_158745	T	G	Mis3
23730	0	9	15874585	chr9_158745	T	G	Mis3
PMis2-NOT-0	1	9	15744298	chr9_157442	A	G	Mis1
B00GG8C	0	9	15874585	chr9_158745	T	G	Mis3
23534	0	9	15874622	chr9_158746	G	T	Mis1
R19-001-00	1	9	15874585	chr9_158745	T	G	Mis3
21032	0	9	15971726	chr9_159717	C	G	Mis1
20765	0	9	15971740	chr9_159717	T	A	Mis2
B00GWAJ	0	9	15971782	chr9_159717	C	T	Mis2
B00FWYX	0	9	15971808	chr9_159718	A	C	Mis2

Gene	BaseChange	AAChange	Transcript	OtherTranscr	MAF	MAFControls	MAFCases
CCDC171	c.737T>G	p.Met246Arg	NM_173550.2		0.0004371	0.0004757	0
CCDC171	c.757C>T	p.Arg253Trp	NM_173550.2		0.0008741	0.0004757	0.005435
CCDC171	c.3524T>G	p.Leu1175Ar	NM_173550.2		0.006993	0.005233	0.02717
CCDC171	c.1064C>G	p.Ala355Gly	NM_173550.2		0.0004401	0.0004794	0
CCDC171	c.1261G>A	p.Val421Met	NM_173550.2		0.0008741	0.0009515	0
CCDC171	c.1261G>A	p.Val421Met	NM_173550.2		0.0008741	0.0009515	0
CCDC171	c.3524T>G	p.Leu1175Ar	NM_173550.2		0.006993	0.005233	0.02717
CCDC171	c.1393T>C	p.Tyr465His	NM_173550.2		0.0004371	0.0004757	0
CCDC171	c.1590A>C	p.Glu530Asp	NM_173550.2		0.0004371	0.0004757	0
CCDC171	c.3873C>G	p.Phe1291Le	NM_173550.2		0.0008749	0.0004762	0.005435
CCDC171	c.2076C>A	p.Asn692Lys	NM_173550.2		0.0004378	0.0004766	0
CCDC171	c.1699C>G	p.Leu567Val	NM_173550.2		0.0004371	0	0.005435
CCDC171	c.757C>T	p.Arg253Trp	NM_173550.2		0.0008741	0.0004757	0.005435
CCDC171	c.3524T>G	p.Leu1175Ar	NM_173550.2		0.006993	0.005233	0.02717
CCDC171	c.2320C>G	p.Gln774Glu	NM_173550.2		0.0004371	0.0004757	0
CCDC171	c.2584C>G	p.Gln862Glu	NM_173550.2		0.0008741	0.0009515	0
CCDC171	c.2584C>G	p.Gln862Glu	NM_173550.2		0.0008741	0.0009515	0
CCDC171	c.2638A>G	p.Met880Val	NM_173550.2		0.0004371	0.0004757	0
CCDC171	c.2692G>A	p.Gly898Arg	NM_173550.2		0.0004371	0.0004757	0
CCDC171	c.3191A>T	p.Gln1064Le	NM_173550.2		0.0004374	0.0004762	0
CCDC171	c.3383A>G	p.Asp1128Gl	NM_173550.2		0.0004371	0.0004757	0
CCDC171	c.3524T>G	p.Leu1175Ar	NM_173550.2		0.006993	0.005233	0.02717
CCDC171	c.2077A>G	p.Met693Val	NM_173550.2		0.0008749	0	0.01087
CCDC171	c.3524T>G	p.Leu1175Ar	NM_173550.2		0.006993	0.005233	0.02717
CCDC171	c.1379G>T	p.Arg460Leu	NM_173550.2		0.0004371	0	0.005435
CCDC171	c.3524T>G	p.Leu1175Ar	NM_173550.2		0.006993	0.005233	0.02717
CCDC171	c.3524T>G	p.Leu1175Ar	NM_173550.2		0.006993	0.005233	0.02717
CCDC171	c.3524T>G	p.Leu1175Ar	NM_173550.2		0.006993	0.005233	0.02717
CCDC171	c.3524T>G	p.Leu1175Ar	NM_173550.2		0.006993	0.005233	0.02717
CCDC171	c.3524T>G	p.Leu1175Ar	NM_173550.2		0.006993	0.005233	0.02717
CCDC171	c.3524T>G	p.Leu1175Ar	NM_173550.2		0.006993	0.005233	0.02717
CCDC171	c.2110delG	p.Glu704fs	NM_173550.2		0.0004371	0	0.005435
CCDC171	c.3524T>G	p.Leu1175Ar	NM_173550.2		0.006993	0.005233	0.02717
CCDC171	c.3524T>G	p.Leu1175Ar	NM_173550.2		0.006993	0.005233	0.02717
CCDC171	c.3524T>G	p.Leu1175Ar	NM_173550.2		0.006993	0.005233	0.02717
CCDC171	c.2077A>G	p.Met693Val	NM_173550.2		0.0008749	0	0.01087
CCDC171	c.3524T>G	p.Leu1175Ar	NM_173550.2		0.006993	0.005233	0.02717
CCDC171	c.3561G>T	p.Met1187Ile	NM_173550.2		0.0004371	0.0004757	0
CCDC171	c.3524T>G	p.Leu1175Ar	NM_173550.2		0.006993	0.005233	0.02717
CCDC171	c.3873C>G	p.Phe1291Le	NM_173550.2		0.0008749	0.0004762	0.005435
CCDC171	c.3887T>A	p.Phe1296Ty	NM_173550.2		0.0004371	0.0004757	0
CCDC171	c.3929C>T	p.Ser1310Ph	NM_173550.2		0.0004371	0.0004757	0
CCDC171	c.3955A>C	p.Asn1319Hi	NM_173550.2		0.0004371	0.0004757	0

ExAC_ALL_AF	ExAC_NFE_AI	ExAC_SAS_AF	ExAC_AMR_A	ExAC_AFR_AI	ExAC_EAS_AF	ExAC_OTH_A	ExAC_FIN_AF
8.248e-06	1.5e-05	0	0	0	0	0	0
3.301e-05	1.501e-05	0	0.0002	0	0.0001	0	0
0.0095	0.0090	0.0271	0.0044	0.0007	0.0002	0.0166	0.0047
0.0001	0.0003	0	0	0	0	0	0
0.0007	0.0009	0.0001	0.0007	0.0005	0	0	0.0006
0.0007	0.0009	0.0001	0.0007	0.0005	0	0	0.0006
0.0095	0.0090	0.0271	0.0044	0.0007	0.0002	0.0166	0.0047
1.67e-05	3.017e-05	0	0	0	0	0	0
7.418e-05	0.0001	0	0	0	0	0	0
0.0005	0.0008	0	0	0	0	0.0011	0.0015
0.0002	0.0002	0.0008	0.0004	0	0	0.0012	0
3.316e-05	.	.	.	.	.	.	.
3.301e-05	1.501e-05	0	0.0002	0	0.0001	0	0
0.0095	0.0090	0.0271	0.0044	0.0007	0.0002	0.0166	0.0047
9.888e-05	0.0001	6.057e-05	8.645e-05	9.619e-05	0	0	0
NA	NA	NA	NA	NA	NA	NA	NA
NA	NA	NA	NA	NA	NA	NA	NA
6.6e-05	0	0	0.0007	0	0	0	0
NA	NA	NA	NA	NA	NA	NA	NA
NA	NA	NA	NA	NA	NA	NA	NA
1.124e-05	2.062e-05	0	0	0	0	0	0
0.0095	0.0090	0.0271	0.0044	0.0007	0.0002	0.0166	0.0047
NA	NA	NA	NA	NA	NA	NA	NA
0.0095	0.0090	0.0271	0.0044	0.0007	0.0002	0.0166	0.0047
NA	NA	NA	NA	NA	NA	NA	NA
0.0095	0.0090	0.0271	0.0044	0.0007	0.0002	0.0166	0.0047
0.0095	0.0090	0.0271	0.0044	0.0007	0.0002	0.0166	0.0047
0.0095	0.0090	0.0271	0.0044	0.0007	0.0002	0.0166	0.0047
0.0095	0.0090	0.0271	0.0044	0.0007	0.0002	0.0166	0.0047
0.0095	0.0090	0.0271	0.0044	0.0007	0.0002	0.0166	0.0047
0.0095	0.0090	0.0271	0.0044	0.0007	0.0002	0.0166	0.0047
NA	NA	NA	NA	NA	NA	NA	NA
0.0095	0.0090	0.0271	0.0044	0.0007	0.0002	0.0166	0.0047
0.0095	0.0090	0.0271	0.0044	0.0007	0.0002	0.0166	0.0047
0.0095	0.0090	0.0271	0.0044	0.0007	0.0002	0.0166	0.0047
NA	NA	NA	NA	NA	NA	NA	NA
0.0095	0.0090	0.0271	0.0044	0.0007	0.0002	0.0166	0.0047
0.0003	0.0005	0	8.708e-05	0.0004	0	0	0
0.0095	0.0090	0.0271	0.0044	0.0007	0.0002	0.0166	0.0047
0.0005	0.0008	0	0	0	0	0.0011	0.0015
8.239e-06	1.499e-05	0	0	0	0	0	0
8.24e-06	1.499e-05	0	0	0	0	0	0
6.599e-05	0.0001	0	0	0	0	0	0

ExAC_maxPO	SIFT	Polyphen2_H	Mutation_tas	pLI score	gTEX_Mean_	gTEX_mean_	gTEX_mean_
1.5e-05	T,T,T	B,B,B,P	N	NA	0.36	0.37	0.36
0.0002	D,D,D	D,P,D,D	N	NA	0.36	0.37	0.36
0.0271	T,T,T,D,.,.	D,D,D,.	D,D	NA	0.36	0.37	0.36
0.0003	T,T	B,B,B	D	NA	0.36	0.37	0.36
0.0009	T,T	B,B,B	D	NA	0.36	0.37	0.36
0.0009	T,T	B,B,B	D	NA	0.36	0.37	0.36
0.0271	T,T,T,D,.,.	D,D,D,.	D,D	NA	0.36	0.37	0.36
3.017e-05	T,T	D,D,D	D	NA	0.36	0.37	0.36
0.0001	T,T	P,P,P	D	NA	0.36	0.37	0.36
0.0015	T	B,B	D	NA	0.36	0.37	0.36
0.0012	T,T	P,P,P	D	NA	0.36	0.37	0.36
3.316e-05	T,T	D,D,D	D	NA	0.36	0.37	0.36
0.0002	D,D,D	D,P,D,D	N	NA	0.36	0.37	0.36
0.0271	T,T,T,D,.,.	D,D,D,.	D,D	NA	0.36	0.37	0.36
0.0001	T,T,T	P,B,B,P	D	NA	0.36	0.37	0.36
NA	T,T,T,.	B,B,B,.	D,D	NA	0.36	0.37	0.36
NA	T,T,T,.	B,B,B,.	D,D	NA	0.36	0.37	0.36
0.0007	T,T,T	B,B,B	D	NA	0.36	0.37	0.36
NA	D,D,D	B,B,B	D	NA	0.36	0.37	0.36
NA	D,D,D,.,.	D,D,D,.	D,D	NA	0.36	0.37	0.36
2.062e-05	D,D,D,.,.	D,D,D,.	D,D	NA	0.36	0.37	0.36
0.0271	T,T,T,D,.,.	D,D,D,.	D,D	NA	0.36	0.37	0.36
NA	T,T	B,B,B	D	NA	0.36	0.37	0.36
0.0271	T,T,T,D,.,.	D,D,D,.	D,D	NA	0.36	0.37	0.36
NA	T,T	B,B,B	D	NA	0.36	0.37	0.36
0.0271	T,T,T,D,.,.	D,D,D,.	D,D	NA	0.36	0.37	0.36
0.0271	T,T,T,D,.,.	D,D,D,.	D,D	NA	0.36	0.37	0.36
0.0271	T,T,T,D,.,.	D,D,D,.	D,D	NA	0.36	0.37	0.36
0.0271	T,T,T,D,.,.	D,D,D,.	D,D	NA	0.36	0.37	0.36
0.0271	T,T,T,D,.,.	D,D,D,.	D,D	NA	0.36	0.37	0.36
0.0271	T,T,T,D,.,.	D,D,D,.	D,D	NA	0.36	0.37	0.36
NA				NA	0.36	0.37	0.36
0.0271	T,T,T,D,.,.	D,D,D,.	D,D	NA	0.36	0.37	0.36
0.0271	T,T,T,D,.,.	D,D,D,.	D,D	NA	0.36	0.37	0.36
0.0271	T,T,T,D,.,.	D,D,D,.	D,D	NA	0.36	0.37	0.36
NA	T,T	B,B,B	D	NA	0.36	0.37	0.36
0.0271	T,T,T,D,.,.	D,D,D,.	D,D	NA	0.36	0.37	0.36
0.0005	T,T,T,T,.,.	B,B,B,.	D,D	NA	0.36	0.37	0.36
0.0271	T,T,T,D,.,.	D,D,D,.	D,D	NA	0.36	0.37	0.36
0.0015	T	B,B	D	NA	0.36	0.37	0.36
1.499e-05	D	B,B	D	NA	0.36	0.37	0.36
1.499e-05	D	B,B	D	NA	0.36	0.37	0.36
0.0001	D	B,B	D	NA	0.36	0.37	0.36

gTEX_Diff_Exp_brain/other
1.018
1.018
1.018
1.018
1.018
1.018
1.018
1.018
1.018
1.018
1.018
1.018
1.018
1.018
1.018
1.018
1.018
1.018
1.018
1.018
1.018
1.018
1.018
1.018
1.018
1.018
1.018
1.018
1.018
1.018
1.018
1.018
1.018
1.018
1.018
1.018
1.018
1.018
1.018
1.018
1.018
1.018
1.018
