# Supplementary material for: Identification of potential genetic risk factors for bipolar disorder by whole-exome sequencing
Source: Transl Psychiatry. 2018 Dec 5;8:268. doi: 10.1038/s41398-018-0291-7 (PMC6281607; doi:10.1038/s41398-018-0291-7)
Supplement: Supplementary file 2 — Supplementary table 2 [file 41398_2018_291_MOESM2_ESM.pdf]

GeneNameFisher	Pathogroup	ncarriers.ca	ncases	ncarriers.cc	ncontrols
ABCC10	PTV + Mis3 + Mis2 + Mis1	15	92	64	1051
ABCC10	Mis3 + Mis2 + Mis1	13	92	60	1051
ACPP	PTV+ Mis3	3	92	0	1051
ACPP	Mis3	2	92	0	1051
ARHGAP9	PTV + Mis3 + Mis2 + Mis1	11	92	36	1051
ARHGAP9	Mis3 + Mis2 + Mis1	11	92	33	1051
BOC	PTV + Mis3 + Mis2 + Mis1	10	92	26	1051
BOC	PTV + Mis3 + Mis2	7	92	13	1051
BOC	Mis3 + Mis2 + Mis1	10	92	26	1051
CCDC171	PTV + Mis3 + Mis2 + Mis1	11	92	31	1051
CCDC171	Mis3 + Mis2 + Mis1	10	92	31	1051
ERI3	PTV + Mis3 + Mis2	3	92	0	1051
ERI3	PTV + Mis3 + Mis2 + Mis1	3	92	0	1051
ERI3	Mis3 + Mis2 + Mis1	3	92	0	1051
FAM19A3	PTV + Mis3 + Mis2 + Mis1	5	92	4	1051
FAM19A3	Mis3 + Mis2 + Mis1	5	92	4	1051
LGR5	PTV + Mis3 + Mis2	10	92	31	1051
LGR5	Mis3 + Mis2	10	92	30	1051
MYO1E	PTV + Mis3 + Mis2	10	92	27	1051
MYO1E	Mis3 + Mis2	10	92	27	1051
NDUFAF2	PTV + Mis3	4	92	2	1051
NDUFAF2	PTV + Mis3 + Mis2	4	92	2	1051
NDUFAF2	PTV + Mis3 + Mis2 + Mis1	4	92	2	1051
NDUFAF2	Mis3 + Mis2 + Mis1	4	92	2	1051
PLCXD3	PTV + Mis3	3	92	0	1051
PLCXD3	Mis3	3	92	0	1051
TCF7L1	PTV + Mis3 + Mis2 + Mis1	5	92	4	1051
TCF7L1	PTV + Mis3 + Mis1	4	92	2	1051
TCF7L1	Mis3 + Mis2 + Mis1	4	92	4	1051
VPS52	PTV + Mis3	5	92	5	1051
VPS52	Mis3	5	92	5	1051

OR	CIinf	CIsup	p.value
3.00	1.51	5.64	9.23E-04
2.71	1.31	5.27	5.61E-03
Inf	4.78	Inf	5.06E-04
Inf	2.16	Inf	6.41E-03
3.82	1.69	8.04	7.75E-04
4.18	1.84	8.87	4.19E-04
4.80	1.99	10.72	3.09E-04
6.55	2.15	18.24	5.68E-04
4.80	1.99	10.72	3.09E-04
4.46	1.95	9.53	2.68E-04
4.00	1.69	8.75	9.68E-04
Inf	4.78	Inf	5.06E-04
Inf	4.78	Inf	5.06E-04
Inf	4.78	Inf	5.06E-04
14.96	3.16	76.78	2.96E-04
14.96	3.16	76.78	2.96E-04
4.00	1.69	8.75	9.68E-04
4.14	1.74	9.09	7.83E-04
4.61	1.92	10.26	3.95E-04
4.61	1.92	10.26	3.95E-04
23.66	3.34	263.93	5.22E-04
23.66	3.34	263.93	5.22E-04
23.66	3.34	263.93	5.22E-04
23.66	3.34	263.93	5.22E-04
Inf	4.78	Inf	5.06E-04
Inf	4.78	Inf	5.06E-04
14.96	3.16	76.78	2.96E-04
23.66	3.34	263.93	5.22E-04
9.47	1.84	44.86	3.62E-03
11.96	2.70	53.13	5.54E-04
11.96	2.70	53.13	5.54E-04
